# Imaging dynamic mTORC1 pathway activity in vivo reveals marked shifts that support time-specific inhibitor therapy in AML

**DOI:** 10.1038/s41467-020-20491-8

**Published:** 2021-01-11

**Authors:** Toshihiko Oki, Francois Mercier, Hiroki Kato, Yookyung Jung, Thomas O. McDonald, Joel A. Spencer, Michael C. Mazzola, Nick van Gastel, Charles P. Lin, Franziska Michor, Toshio Kitamura, David T. Scadden

**Affiliations:** 1 Center for Regenerative Medicine, Massachusetts General Hospital, Boston, MA 02114 USA; 2 Department of Stem Cell and Regenerative Biology, Harvard University, Cambridge, MA 02138 USA; 3 Harvard Stem Cell Institute, Harvard University, Cambridge, MA 02138 USA; 4 Center for Systems Biology and Wellman Center for Photomedicine, Massachusetts General Hospital, Boston, MA 02114 USA; 5 Center for Cancer Evolution and Department of Data Sciences, Dana-Farber Cancer Institute, Boston, MA 02215 USA; 6 Department of Biostatistics, Harvard T.H. Chan School of Public Health, Boston, MA 02215 USA; 7 The Broad Institute of Harvard and MIT, Cambridge, MA 02139 USA; 8 The Ludwig Center at Harvard, Boston, MA 02215 USA; 9 Division of Cellular Therapy, The Institute of Medical Science, University of Tokyo, 108-8639 Tokyo, Japan; 10 Present Address: McGill University, Montreal, QC H3A 0G4 Canada; 11 Present Address: School of Engineering, University of California, Merced, Merced, CA 95343 USA

**Keywords:** Fluorescence imaging, Chemotherapy, Acute myeloid leukaemia

## Abstract

Acute myeloid leukemia (AML) is a high remission, high relapse fatal blood cancer. Although mTORC1 is a master regulator of cell proliferation and survival, its inhibitors have not performed well as AML treatments. To uncover the dynamics of mTORC1 activity in vivo, fluorescent probes are developed to track single cell proliferation, apoptosis and mTORC1 activity of AML cells in the bone marrow of live animals and to quantify these activities in the context of microanatomical localization and intra-tumoral heterogeneity. When chemotherapy drugs commonly used clinically are given to mice with AML, apoptosis is rapid, diffuse and not preferentially restricted to anatomic sites. Dynamic measurement of mTORC1 activity indicated a decline in mTORC1 activity with AML progression. However, at the time of maximal chemotherapy response, mTORC1 signaling is high and positively correlated with a leukemia stemness transcriptional profile. Cell barcoding reveals the induction of mTORC1 activity rather than selection of mTORC1 high cells and timed inhibition of mTORC1 improved the killing of AML cells. These data define the real-time dynamics of AML and the mTORC1 pathway in association with AML growth, response to and relapse after chemotherapy. They provide guidance for timed intervention with pathway-specific inhibitors.

## Abstract (web summary)

The role of mTORC1 in AML has not yet been proven due to the mixed results of its inhibitors in clinical trials. Here the authors show the real-time dynamics of the mTORC1 pathway in association with AML growth and response to chemotherapy with fluorescent markers, providing guidance for timed intervention with pathway-specific inhibitors.

## Introduction

Acute myeloid leukemia (AML) is a highly lethal hematological malignancy. Approximately 20,000 people are newly diagnosed per year in the U.S. and more than half of them ultimately die of the disease^1^. Although conventional chemotherapies can induce remission, the majority of patients experience relapse of the disease. Therefore, identifying targetable vulnerabilities of AML following standard chemotherapy is warranted.

mTORC1 signaling regulates the proliferation and survival of cells in response to environmental changes in nutrients, growth factors, and oxygen^2–4^. It is a master regulator of cell growth and is widely reported to be a critical pathway in cancer cells including AML^2,3^. mTORC1 inhibition has been reported to abrogate the progression of malignancies including AML in vitro and in vivo^3,5–8^. In addition, recurrent mutations, including *FLT3-ITD* and other tyrosine kinases, can result in activation of mTORC1 signaling, making it an attractive target for AML treatment rather than targeting each specific mutation^9,10^. Therefore, mTORC1 inhibition has been considered for potential treatment strategies for AML^3,7,11,12^, but clinical use of mTORC1 inhibitors has shown limited efficacy^8,12^.

Since mTORC1 activity depends on growth signals and nutrient availability in the microenvironment^13–16^, it is likely that mTORC1 activity changes dynamically depending on cell anatomical location and, perhaps, the dramatic environmental shifts accompanying chemotherapy.

In this study, we seek to monitor mTORC1 activity over time in live animals, reasoning that mTORC1 activity may be very different depending on the in vivo context of cells. Combining intravital imaging and a dynamic probe of mTORC1 activity during growth, treatment and relapse of an AML model in mice, we define distinct temporal features of mTORC1 activity that argue for time-specific targeting of it.

## Results

### Development of a dynamic mTORC1 probe

**Fig. 1:**
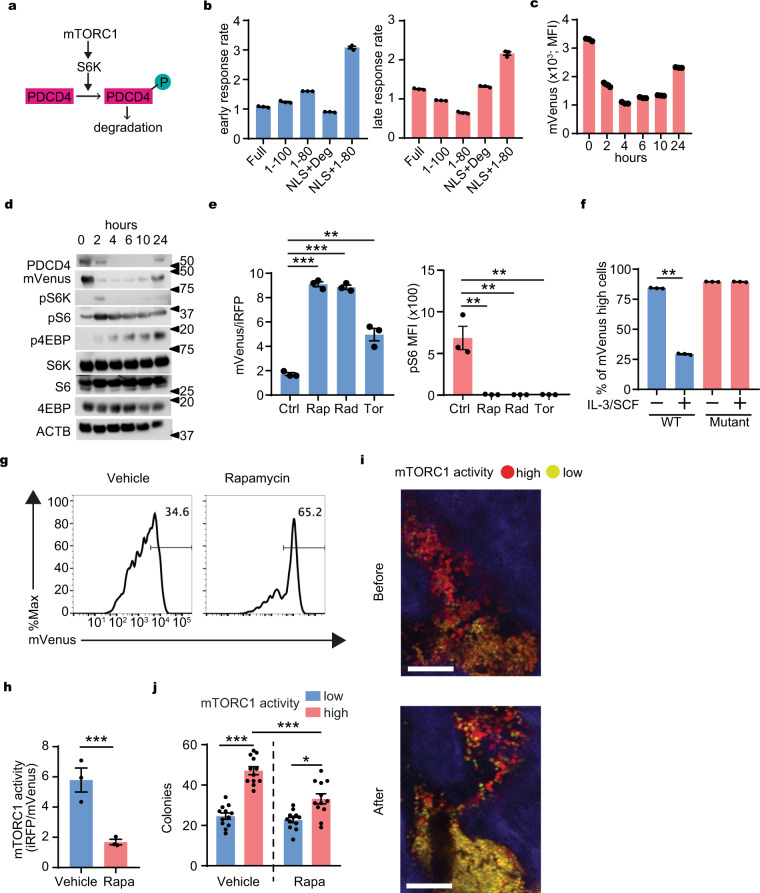
Development of mTORC1 probe. **a** A schematic model of PDCD4 degradation under mTORC1 signal. **b** Ratio (response rate) of the green fluorescence intensity of NIH3T3 cells transduced with mVenus fused to full length, partial fragments, or degron (Deg) fragment of PDCD4 with or without SV40NLS (Full, 1–100, 1–80, NLS + Deg, NLS + 1–80) at 2 h (early) and 4 h (late) after serum re-addition compare to the intensity at 0 h (*n* = 3). **c** Changes in intensity of mVenus-TOSI in NIH 3T3 at indicated times after serum readdition (*n* = 3). **d** Representative changes in abundance of PDCD4, mVenus-TOSI, and phosphorylation of mTORC1 targets at indicated times after serum re-addition in NIH 3T3. **e** The effect of mTORC1 inhibitors on mVenus intensity (left) and phosphor-S6 (right) in murine AML cells. Rapamaycin (Rap), Rad001 (Rad), Torin II (Tor) (*n* = 3). **f** Response of the mVenus probes with or without mutation in the phosphorylation sites of PDCD4 in murine AML cells (*n* = 3). **g** Representative histogram plot of mVenus intensity in AML cells harvested from mice treated with vehicle or rapamycin. **h** mTORC1 activity of harvested AML cells from mice with vehicle or rapamycin (Rapa) treatment (*n* = 3). **i** Representative images of in vivo imaging with a 2-photon microscope of the calvarial bone marrow before and 24 h after rapamycin treatment (4 mg/kg i.p.) from a similar visual field from same mouse. Red (mVenus^−^/TdTomato^+^): mTORC1 high cells, Yellow (mVenus^+^/TdTomato^+^): mTORC1 low cells. Scale bar = 100 μm. **j** Clone forming capacity of mTORC1 high and low cells harvested from the mice (described in **g** and **h**) treated with vehicle or rapamycin (Rapa) (*n* = 12). Mean ± SEM was shown in bar pot (each dot represents each sample). **b**–**f** Representative data from at least three independent experiments were shown. **g**–**j** Representative data from at least two independent experiments were shown. Statistical analysis was performed by using two-way *t*-test (**f**, **h**) or paired *t*-test (**e**, **j**) (*p < 0.05, **p < 0.01, ***p < 0.001).

To monitor mTORC1 activity, we developed a real-time indicator of mTORC1 activity. Programmed cell death 4 (PDCD4) is a ubiquitously expressed nuclear localization signal (NLS)-containing protein and a downstream target of mTORC1^2^. Once mTORC1 is activated, PDCD4 is rapidly phosphorylated by S6 kinase (S6K), ubiquitinated and degraded by the proteasome^2,17^. (Fig. 1a). Therefore, abundance of PDCD4 can be used as a negative indicator of mTORC1 activity.

First, we made retroviral constructs for fusion proteins composed of coding sequences for mVenus fluorescent protein and full-length or multiple deletion mutants of PDCD4 that contain its degron (a.a. 62–76)^17^, with or without a nuclear localization signal (NLS). These were evaluated in the mouse fibroblast cell line NIH 3T3 in which PDCD4 is rapidly degraded after induction of mTORC1 signaling^17^. We assessed the different fusion constructs after serum refeeding to starved NIH 3T3 for induction of mTORC1 activity. Among these transfectants, NLS with the N-terminal 80 amino acids of PDCD4 (NLS+1–80) appeared to be the most sensitive probe for mTORC1–activity (Fig. 1b, and Supplementary Fig. 1a). Therefore, this probe was used in the rest of this study.

The inverse relationship of mVenus signal intensity to mTORC1 activity was demonstrated by flow cytometry and western blot analysis of phosphorylated downstream targets of mTORC1 over time following serum refeeding of the transduced cells (Fig. 1c, d). The mVenus signal intensity dynamically shifted from high to low and back to baseline within 24 h, indicating its dynamic sensitivity as a reporter of mTORC1 activity. Next, inhibitors of mTORC1, including rapamycin were added to serum refed cells after serum starvation. As predicted, the mVenus signal intensity remained high in the presence of mTORC1 inhibitors whose active suppression of mTORC1 was shown by pS6 levels compared to control (Fig. 1e and Supplementary Fig. 1b). mVenus intensity correlated with pS6 levels confirming the accuracy of this probe as a monitor of mTORC1 activity (Supplementary Fig. 1c). On the other hand, the PDCD4 mutant possessing site directed mutations at two S6K target phosphorylation sites in PDCD4 (PDCD4 mutant probe; mVenusNLS-PDCD4S67AS71A) (1–80) lacked sensitivity to mTORC1 activity (Fig. 1f). These data confirm that the signal intensity depends upon PDCD4 phosphorylation by mTORC1. In addition, the utility of the mVenusNLS-PDCD4 (1–80) probe was further confirmed in other cell lines including mouse and human AML cell lines (Supplementary Fig. 1d). These data indicate that mVenusNLS-PDCD4 (1–80) persistence is inversely correlated with mTORC1 activity and quickly responds to changes in mTORC1 activity in cultured cells. The probe was hereafter entitled mVenus-TOSI (TOr-Signal-Indicator).

To further confirm that our PDCD4-derived degron based probe responded to mTORC1 signaling, we developed a similar mCherry based PDCD4 degron containing the TOSI probe (mCherry-TOSI). This probe allowed us to analyze mTORC1 activity in GFP-expressing cells and measured its response to knocking out *Raptor*. We transduced mCherry-TOSI or control mCherry into CreERT-*Raptor*^fl/fl^ AML cells and induced *Raptor* knock-out by adding hydroxytamoxifen (HTM). This resulted in an increase of mCherry-TOSI without affecting the WT control cells (Supplementary Fig. 1e). In addition, we also co-expressed constitutively active S6K (S6KCA) and mCherry-TOSI in mouse MLL-AF9 AML cells and observed the anticipated reduction in mVenus (Supplementary Fig. 1f). These experiments confirm that the probe was reflective of changes in the mTORC1 signaling pathway.

### mTORC1 activity declines during AML progression in vivo

To evaluate mTORC1 activity in a mouse model of AML, we used mVenus-TOSI in the context of cells bearing the potent leukemogenic fusion MLL-AF9. We retrovirally transduced mVenus-TOSI into a mouse AML cell line (FM4) that expresses the retrovirally transduced MLL-AF9 oncogene and iRFP. From these, several single cell derived clones were generated to assure uniformity of mVenus signal intensity. mVenus-TOSI transduced clones with the brightest mVenus signal intensity were used for further experiments (FM4-mVenus). For in vivo imaging, we also retrovirally transduced TdTomato fluorescent protein, which produces a much brighter signal than iRFP, into FM4-mVenus cells and made single cell clones expressing both fluorophores (FM4-mVenus-TdTomato; FM4-VT). As expected from prior reports^18^, transplantation of the cell lines FM4, FM4-mVenus or FM4-VT into sublethally irradiated mice induced AML which was lethal in all recipients within 2 months after transplantation.

To test if mVenus-TOSI detects changes in mTORC1 activity in vivo, we evaluated mVenus using FM4-VT with or without rapamycin treatment one week after AML cell transplantation. As expected, mVenus was increased in response to rapamycin treatment (Fig. 1g and Supplementary Fig. 1g) and the iRFP:mVenus ratio decreased (Fig. 1h). By in vivo imaging, mTORC1 activity in the same region of the mouse calvarial bone marrow could be tracked over time, and the evolution of mTORC1 high (red; mVenus^−^/TdTomato^+^) and mTORC1 low (yellow; mVenus^+^/TdTomato^+^) populations could be visualized. As shown in Fig. 1i and Supplementary Fig. 1h, rapamycin treatment resulted in decreased mTORC1 activity (mVenus^+^/TdTomato^+^) similar to the changes observed using flow cytometry. Therefore, the probe is useful for assessing mTORC1 activity in isolated cells or in vivo. Cells with high mTORC1 activity showed higher colony-forming capacity compared to cells with low mTORC1 activity (Fig. 1j), indicating that changes in mVenus intensity reflect biologically important alterations in mTORC1 signaling. Furthermore, cells with indicator levels of higher mTORC1 activity were more sensitive to rapamycin (Fig. 1j), consistent with graduated indication of the mTORC1 pathway at levels meaningful for cell function.

**Fig. 2:**
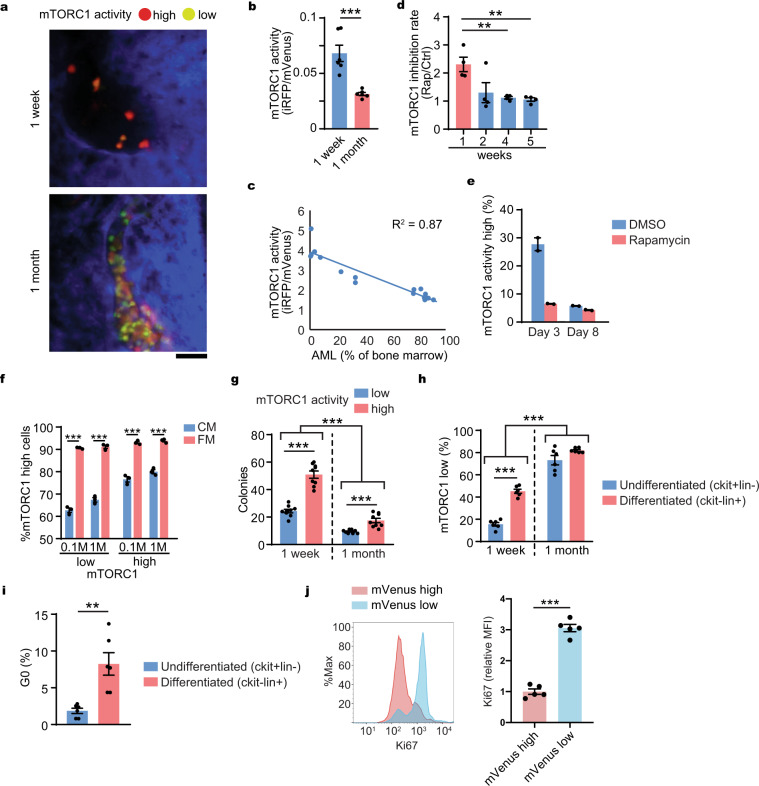
mTORC1 activity is downregulated during AML progression. **a** Representative in vivo imaging of AML cell with mVenus-TOSI probe (FM4-Venus-TdT) in the mouse calvarial bone marrow from a similar visual field of the same mouse using a confocal microscope during disease development at 1 week and 1 month after transplantation. Scale = 50 μm. Red (mVenus^−/^TdTomato^+^): mTORC1-high cells, Yellow-Green (mVenus^+^/TdTomato^+^): mTORC1-low cells. **b** Flow cytometry analysis of mVenus-TOSI probe intensity during disease development at 1 week and 1 month after transplantation. The AML cells were harvested from femurs of the mice (*n* = 6/time point). **c** Correlation between AML burden and mTORC1 activity in AML cells with mVenus-TOSI probe (FM4-Venus) (*n* = 16). **d** mTORC1 inhibition rate (mVenus intensity of AML cells from rapamycin treated mice/mVenus intensity of AML cells from vehicle treated mice) by a single rapamycin treatment (4 mg/kg i.p.) at different time points after transplantation. The AML cells were harvested from femurs of the mice (*n* = 4/time point/treatment). **e** mTORC1 activity in AML cells cultured in vitro obtained at indicated days after starting rapamycin treatment (*n* = 2). **f** Proportions of mTORC1-high cells cultured at high (1 M/ml) and low (0.1 M/ml) concentrations with after 24 h in conditioned medium (CM) or fresh medium (FM) (*n* = 3). **g** Colony-forming capacity of mVenus-low and high AML cells harvested at early (1 week) or late (1 month) time points after transplantation (*n* = 9). **h** Proportions of mTORC1-low cells in undifferentiated or differentiated AML cells at different time points after transplantation (*n* = 6). **i** Proportions of G0 phase (mVenus high population) of AML cells (FM4-mVenus-p27K^−^) in undifferentiated or differentiated AML cells after transplantation (*n* = 6). **j** Cell cycle analysis of AML cells 1 week after transplantation. Ki67 staining intensity of mVenus high or low cells were analyzed by flow cytometry (*n* = 5). **a**–**j** Representative data from at least two independent experiments were shown. Mean ± SEM was shown in bar pot (each dot represents each sample). Statistical analysis was performed by using two-way *t*-test (**b**, **f**, **i**, **j**) or paired *t*-test (**d**, **g**, **h**) (**p < 0.01, ***p < 0.001).

To monitor mTORC1 signal dynamics during AML development, mVenus was tracked over time by in vivo imaging after transplantation of FM4-VT (Fig. 2a, and Supplementary Fig. 2a, b). The observed shift from red to yellow was consistent with mTORC1 activity gradually decreasing as AML progressed. Correspondent changes were evident when cells were isolated and analyzed by flow cytometry (Fig. 2b). Interestingly, there was a bimodal peak at 1 week (Supplementary Fig. 2c upper panel and Supplementary Fig. 2d left) that disappeared 1 month after transplantation (Supplementary Fig. 2c lower panel and Supplementary Fig. 2d right), indicating the existence of heterogenous mTORC1 activity that is globally repressed over time with AML progression and negatively correlated with AML burden in the bone marrow (Fig. 2c).

We found that rapamycin only affected cells at 1 week (Fig. 2d). Similar to in vivo, the AML cells cultured with longer time (day 8) had less mTORC1 activity than the AML cells cultured with shorter time (day 3) in vitro (Fig. 2e). The basis for this observation is unclear but we hypothesized that the limiting availability of nutrients, growth factors and oxygen accompanying AML progression likely contributes. We assessed whether cell density alone or nutrient availability affected mTORC1 activity by comparing mTORC1 activity of high density (1 million cells/ml) and low density (0.1 million cells/ml) AML cells cultured with conditioned or fresh medium. We found that the replacement of cell culture medium with fresh medium (and therefore nutrient availability) was a greater determinant of mTORC1 activity than cell density (Fig. 2f). Using in vivo imaging to define anatomic relationships of AML cells to bone structures, we found that mTORC1 activity was associated with the distance from bone (Supplementary Fig. 2e), further demonstrating that environmental factors influence mTORC1 activity. Colony formation was assessed using mTORC1 low and high populations harvested at 1 week or 1 month after transplantation. Colony formation was highest among mTORC1 high AML cells and the cells harvested 1 week after transplantation (Fig. 2g). Colony forming capacity is reported to correlate with leukemia-initiating capacity^19^ and in keeping with this, we found that mTORC1 activity (Fig. 2h and Supplementary Fig. 2f–g) and cell cycling were higher in undifferentiated leukemic cells (Fig. 2i). Furthermore, we confirmed that mTORC1 high AML cells were more actively cycling than mTORC1 low AML cells at 1 week after transplantation in vivo (Fig. 2j). Therefore, mTORC1 activity is variable in association with cell properties and is dynamically altered in vivo in response to disease state. Correspondingly, the sensitivity to mTORC1 inhibition will likely depend on the extent and localization of disease.

### mTORC1 activity is heterogeneous within AML

**Fig. 3:**
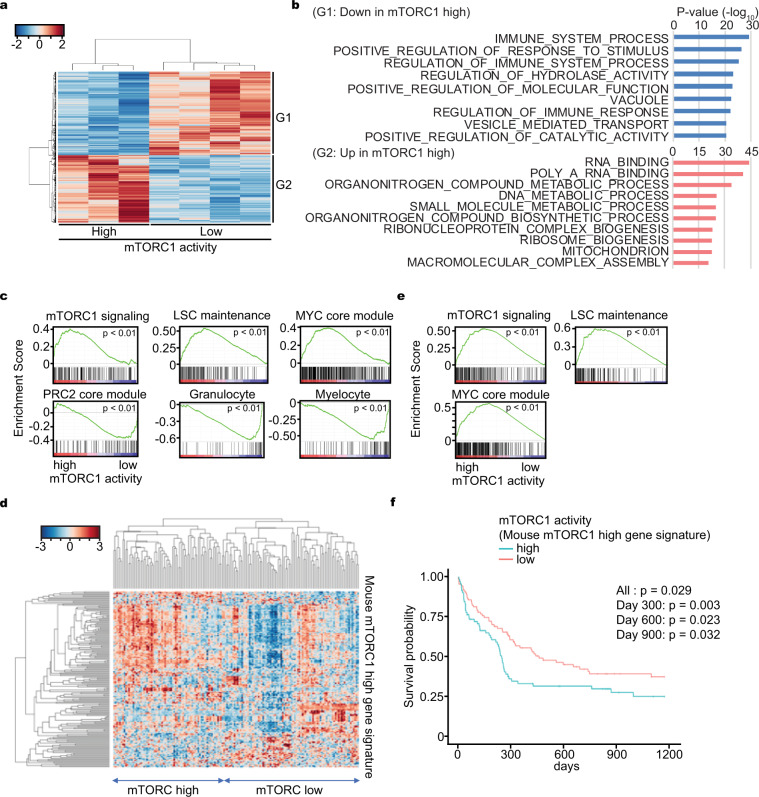
Expression profiling between mTORC1 high and low AML cells. **a** Clustering analysis of transcriptomic data obtained from mTORC1 high or low AML cells harvested from mouse bone marrow. **b** Gene ontology analysis of differentially expressed genes obtained from mTORC1 high or low AML cells harvested from mouse bone marrow. **c** Gene set enrichment analysis (GSEA) of differentially expressed genes obtained from mTORC1 high or low AML cells harvested from mouse bone marrow. **d** Classification of human AML samples (GSE12417) with the mouse mTORC1 high AML cell gene signature (mouse mTORC1 high gene signature). **e** GSEA of transcriptomic data (GSE12417) obtained from human AML samples classified according to their expression of the mouse mTORC1 high gene signature. **f** Overall survival probability of AML patients classified according to their expression of the mouse mTORC1 high gene signature. Overall log-rank test *p*-value (All) and the *p*-value at different time points (day 300, 600, and 900) were shown.

To further assess the heterogeneity of mTORC1 activity observed in vivo, we performed RNA-seq on mTORC1-high and low AML cells sorted by mVenus intensity. The transcriptomic profiles were distinctive (Fig. 3a) and gene ontology analysis revealed that mTORC1 activity was associated with differentially expressed immune response and metabolism related genes (Fig. 3b). Further, gene signature enrichment analysis (GSEA) validated that the mTORC1 signal signature correlated with mTORC1 activity and that leukemia stem cell (LSC)^20^, Myc^21^, and cell cycle^22–24^ related signatures were enriched in mTORC1 high cells (Fig. 3c and Supplementary Fig. 3a). Conversely, PRC2^21^, granulocyte^25^, and myeloid differentiation^25^ signatures were enriched in mTORC1 low cells (Fig. 3c). These data are consistent with mTORC1 high cells being proliferative and transcriptionally related to LSC under specific environmental conditions^6,26,27^.

To determine whether the findings in the mouse AML model had relevance to human disease, we performed unsupervised hierarchical clustering analysis on public data composed of 163 primary adult human AML samples (GSE12417)^28^ depending on the expression of highly expressed genes (mouse mTORC1 high gene signature (mTOR-GS)) in mTORC1 high compared to mTORC1 low mouse AML cells. The human AML samples were stratified into two distinct clusters by the differential expression of the mouse mTORC1 high gene signature (Fig. 3d). Further, GSEA on human AML with high and low expression of the mouse mTORC1 high gene signature revealed that mTORC1 activity positively correlated with LSC and Myc signatures as observed in our mouse model (Fig. 3e). Interestingly, we also found that human AML patients with higher expression of the mouse mTORC1 high gene signature had a poorer prognosis (Fig. 3f). In addition, similar analysis with mTORC1 target gene sets from the Molecular Signature Database (MSigDB) revealed that higher expression of mTORC1 target genes were heterogeneous between AML cases and negatively correlated with prognosis (Supplementary Fig. 3b–d). Furthermore, we compared highly expressed genes (log_2_ fold change > 0.5 and FDR < 0.05) in mTORC1 high AML patients (2800 genes) (human mTOR-GS) with those in mouse mTOR-GS (158 genes) and found that 44 genes overlapped (overlapping *p*-value = 0.022). Pathway analysis on these 44 overlapped genes in MSigDB revealed Myc and mTORC1 related signatures were enriched (Supplementary Fig. 3e). Therefore, the core component of mTORC1 high/low AMLs are similar between mouse and human and related to Myc signaling. We also analyzed single cell RNA-seq data of AML from a publicly available data set (GSE116256)^29^ and found there was intratumor heterogeneity in mTORC1 signaling in some AML cases (Supplementary Fig. 3f, g). Therefore, mTORC1 activity is heterogeneous among AML cases and within an individual AML case, which is similar to our observations in the mouse. In our experimental context, high mTORC1 activity was associated with an LSC signature and limited differentiation. In the clinical context, a higher mTORC1 signature is indicative of poor prognosis.

### mTORC1 signal dynamics during response to chemotherapy

**Fig. 4:**
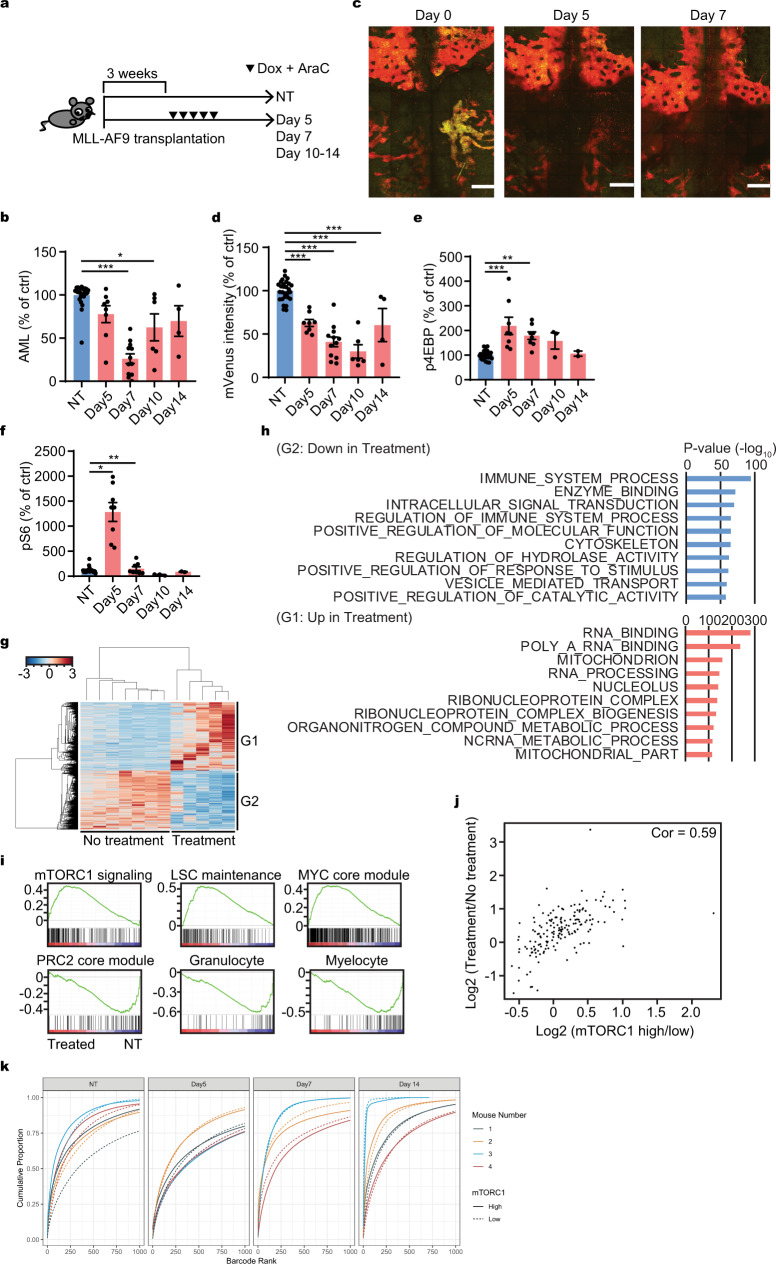
mTORC1 activity dynamics during chemotherapy. **a** Experimental workflow of bone marrow harvest in relationship to chemotherapy administration (day 0: first day of treatment). **b** Relative of AML burden without (NT) or after chemotherapy (day 5, 7, 10, and 14). The numbers were normalized to the values at NT (*n* = 28, 8, 12, 6, 4, respectively). **c** Representative intravital imaging of z-projection of the entire calvarial bone marrow with a 2-photon microscope of a FM4-Venus/TOSI transplanted mouse before (day 0) and after chemotherapy (day 5 and 7). Red (mVenus^−^/TdTomato^+^): mTORC1 high area, Yellow (mVenus^+^/TdTomato^+^): mTORC1 low area. Multiple z-projection images are stitched together to show a large area of the calvarial bone marrow. Scale = 500 μm. **d** Relative mVenus intensity by flow cytometry of FM4-mVenus-TOSI cells in murine recipient without (NT) or after chemotherapy (day 5, 7, 10, and 14). The numbers were normalized to the average value at NT (*n* = 28, 8, 12, 6, 4, respectively). **e** Relative p4EBP during chemotherapy without (NT) or after chemotherapy (day 5, 7, 10, and 14) by flow cytometry. The numbers were normalized to the average value at NT (*n* = 26, 8, 8, 3, 2, respectively). **f** Relative pS6 during chemotherapy without (NT) or after chemotherapy (day 5, 7, 10, and 14) by flow cytometry. The numbers were normalized to the average value at NT (*n* = 26, 8, 8, 3, 2, respectively). **g** Clustering analysis of the differentially expressed genes obtained from AML cells harvested from mouse bone marrow with or without chemotherapy (day 7). **h** GO analysis of the differentially expressed genes obtained from AML cells harvested from mouse bone marrow with or without chemotherapy (day 7). **i** GSEA of differentially expressed genes obtained from AML cells harvested from mouse bone marrow with or without chemotherapy (day 7). **j** Correlation between the differentially expressed genes between with or without chemotherapy and those between mTORC1 high and low cells (analyzed in Fig. 3a). **k** Clonal diversity before and after chemotherapy. Barcodes are ordered by decreasing proportion and cumulative proportion was plotted. Steeper curves indicate higher levels of selection due to less barcodes representing a larger proportion of the population. mTORC1 high (solid line) and mTORC1 low (dashed line) barcodes come from the same experiment, so differences between the curves indicate selection for one relative to the other. For the technical detail about this analysis, see “Methods” section. **b**, **d**–**f** Pooled data from at least two independent experiments were shown. **c** Representative data from two independent experiments were shown. Statistical analysis was performed by using two-way paired *t*-test (*p < 0.05, **p < 0.01, ***p < 0.001).

Having identified that mTORC1 activity is related to LSC-like function and to AML prognosis, we assessed correlations between mTORC1 activity and sensitivity to cytotoxic chemotherapy. First, we evaluated sensitivity to the cell cycle specific nucleoside analog cytosine arabinoside (Ara-C) in vitro. Ara-C selectively killed the mTORC1 high cells in vitro (Supplementary Fig. 4a). Next, we measured AML burden and mTORC1 activity at different time points. After treatment of FM4-mVenus or FM4-VT AML bearing mice with chemotherapy drugs doxorubicin and AraC (Fig. 4a) similar to the regimen used for human patients^18,30^, we found that the AML burden was reduced with the nadir at ~day 7 (Fig. 4b). Unexpectedly, we observed a notable reduction of mTORC1 low cells by both intravital microscopy observation (Fig. 4c and Supplementary Fig. 4b–d) and by flow cytometry (Fig. 4d). In addition, assessment of mTORC1 activity by phospho-flow showed increased phosphorylation of both 4EBP and S6 after chemotherapy (Fig. 4e, f). Therefore, mTORC1 signaling became higher after chemotherapy in vivo, in contrast to the in vitro observation (Supplementary Fig. 4a).

Gene expression analysis on day 7 of AML cells with or without chemotherapy identified clearly distinguishable transcriptomic profiles (Fig. 4g). GO analysis revealed that gene alterations after chemotherapy were related to immune response, RNA biology and several metabolomic processes (Fig. 4h). As might be anticipated, these differences in GO with or without chemotherapy were similar to those seen in untreated AML with high and low mTORC1 activity (Fig. 3b). Consistent with this observation, mTORC1 signal-related GSEA signatures were enriched in AML cells after chemotherapy including those of stemness and impaired differentiation (Fig. 4i). The transcriptomic changes between treatment and no treatment highly correlated with the changes between mTORC1 low and high cells (Fig. 4j). In addition, we revisited the sets of differentially expressed genes after chemotherapy in human AML as defined by others^31,32^. Gene-set overlap analysis with MsigDB showed a significant overlap between the gene sets highly expressed in AML after chemotherapy and the mTORC1 signal related gene set (Tables 1, 2) further confirming correspondence between the mouse model and human AML. Interestingly, GSEA analysis also showed that cell cycle related gene signatures enriched after chemotherapy (Supplementary Fig. 4e). In line with this notion, chemotherapy indeed increased mTORC1 activity and cell cycle in the remaining cells (Supplementary Fig. 4f).

**Table 1:** Gene set enriched in human AML sample after chemotherapy.

Gene Set name	FDR *q*-value
HALLMARK_UV_RESPONSE_DN	1.31E−06
HALLMARK_EPITHELIAL_MESENCHYMAL_TRANSITION	7.38E−05
HALLMARK_IL2_STAT5_SIGNALING	7.38E−05
HALLMARK_COAGULATION	7.93E−05
HALLMARK_PROTEIN_SECRETION	1.61E−04
HALLMARK_ANDROGEN_RESPONSE	2.56E−03
HALLMARK_APICAL_JUNCTION	2.56E−03
HALLMARK_COMPLEMENT	2.56E−03
HALLMARK_KRAS_SIGNALING_UP	2.56E−03
**HALLMARK_MTORC1_SIGNALING**	**2.56E−03**
HALLMARK_APOPTOSIS	8.77E−03
HALLMARK_MITOTIC_SPINDLE	1.62E−02
HALLMARK_XENOBIOTIC_METABOLISM	1.62E−02
HALLMARK_TGF_BETA_SIGNALING	4.78E−02

Gene set overlap analysis of the gene set highly expressed in human AML samples described by Hackl et al.^32^ with Hallmarks gene sets from MsigDB was performed.

Entries related to mTORC1 signal are highlighted in bold.

**Table 2:** Gene set enriched in human drug resistant AML after chemotherapy.

Gene Set name	FDR *q*-value
HALLMARK_ALLOGRAFT_REJECTION	3.43E−16
**HALLMARK_MTORC1_SIGNALING**	**1.18E−11**
HALLMARK_TNFA_SIGNALING_VIA_NFKB	1.18E−11
HALLMARK_IL2_STAT5_SIGNALING	1.49E−09
HALLMARK_INFLAMMATORY_RESPONSE	1.49E−09
HALLMARK_IL6_JAK_STAT3_SIGNALING	1.19E−08
HALLMARK_COMPLEMENT	1.19E−08
HALLMARK_KRAS_SIGNALING_UP	1.19E−08
HALLMARK_INTERFERON_GAMMA_RESPONSE	1.33E−06
HALLMARK_ADIPOGENESIS	1.05E−05

Gene-set overlap analysis of the gene set highly expressed in drug resistant human AML samples described by Saito et al.^31^ with Hallmarks gene sets from MsigDB was performed.

Entries related to mTORC1 signal are highlighted in bold.

There are two possible mechanisms for the shift of gene expression to a high mTORC1 activity signature after chemotherapy; one is the selection of mTORC1 high cells and the second is the induction of mTORC1 activity following chemotherapy. We assessed these two possible scenarios by introducing genetic barcodes using the ClonTracer library^33^ that allowed for clonal tracking of ~0.2 million AML cells. The barcoded cells were transplanted into recipient mice. They were harvested before and at different time points after chemotherapy (day 5, 7, and 14). Cells were sorted based on their apparent mTORC1 activity and clonal diversity was measured by DNA sequencing. We reasoned that selection would be accompanied by a more restricted pool of barcodes after chemotherapy^33^. Selection by chemotherapy should cause limited clonal abundance in the mTORC1 high population compared to the mTORC1 low population. In contrast, induction would result in relatively constant representation of clones before and after chemotherapy since in that case, barcodes could exist in and transition between each state, eventually resulting in a stationary barcode distribution.

We found that a significant proportion of unique barcodes are represented in both the mTORC1 high and low cell populations (Supplementary Fig. 4g). Since barcodes only infect a single cell which has to exist in a high or low state, a significant proportion of barcodes that are represented by cells in both states suggests that induction occurs. Otherwise the proportion should be close to an extreme (0 or 1) meaning most of the populations exist only in mTORC1 high or mTORC1 low states. In addition, clonal abundance based on barcodes revealed more similarity with respect to the cumulative proportion of represented barcodes between mTORC1 high and low cells in treated cohorts compared with non-treated cohorts. These data suggest a more similar distribution of mTORC1 high and low cells in these populations which would arise primarily due to induction rather than selection (Fig. 4k). Among barcodes that were represented by mTORC1 high and low cells, the distribution of the log-odds ratio of mTORC1 high cells to mTORC1 low cells was symmetric and centered around 0, indicating a similar number of mTORC1 high and low cells within a barcode (Fig. 4k and Supplementary Fig. 4h). Collectively, these data point to mTORC1 induction and not mTORC1 high cell selection by chemotherapy.

We next sought to define whether or not the cell death occurred in a location-dependent manner during chemotherapy, since mTORC1 activity is responsive to environmental conditions. To this end, we developed AML cells stably expressing a caspase3-sensitive FRET probe (FM4-SCAT3.2) and tested it in our AML treatment model. This probe is a modified version of an established FRET probe with a caspase-3 sensitive DEDV sequence, SCAT3. When excited near the peak of ECFP excitation (e.g., 435 nm for one photon and 860–870 nm for two photons), the SCAT3 probe emits mVenus or YFP (FRET) signal when it is intact, but emits ECFP signal (no FRET) when its DEDV sequence is cleaved^34^. However, the SCAT3 probes to date have had limited utility because of frequent recombination of the two jellyfish fluorescent proteins^35^. We have overcome this limitation through the use of Amcyan, a cyan fluorescent protein derived from a coral and making an Amcyan version of SCAT3 (SCAT3.2) (Supplementary Fig. 5a). This substance can be used as a compatible fluorophore with ECFP and does not have any apparent issues of recombination^35,36^. In vitro time lapse imaging of SCAT3.2 AML cells revealed that Ara-C treatment indeed induced apoptosis of individual cells in as short as 40 min (Supplementary Fig. 5b and Movie 1). Since these changes were not observed in controls (Supplementary Fig. 5b) and because of the close temporal association with chemotherapy, we conclude that the changes in FRET are due to chemotherapy. We then used the SCAT3.2 AML cells in the transplantation and treatment model shown in Fig. 4a. Flow cytometric analysis showed that maximal apoptosis occurred at the beginning of chemotherapy (day 2) and returned to baseline by the time of the cell nadir at day 6 (Supplementary Fig. 5c). Therefore, we chose day 2 of chemotherapy to assess apoptosis in vivo by intravital imaging. Consistent with what was observed in vitro, apoptosis was evident at 75 min by in vivo time lapse imaging (Supplementary Fig. 5d and Movies 2 and 3). The onset of apoptosis was more rapid (min) compared to mTORC1 alterations (days), suggesting that chemotherapy-induced apoptotic process per se was not the direct cause of differential mTORC1 activity during chemotherapy. Notably, no clear relationship of apoptosis to anatomic landmarks was noted (Supplementary Fig. 5e, f), supporting the notion that mTORC1 high cells were not selected by chemotherapy due to their anatomic location.

### Timed inhibition of the mTORC1 pathway alters the outcome of chemotherapy

**Fig. 5:**
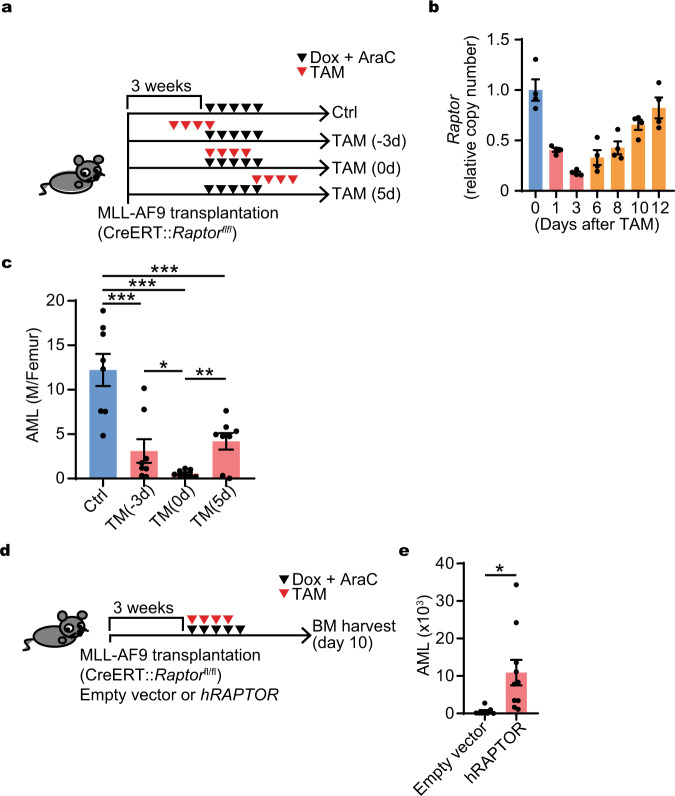
Timed inhibition of mTORC1 with chemotherapy improved AML burden. **a** Experimental design of mTORC1 timed inhibition in combination with chemotherapy. MLL-AF9 cells (GPF^+^, CreERT::Rptor^fl/fl^) were transplanted 3 weeks before the start of chemotherapy (Dox + Ara-C), which was started day 0 with (TAM) or without (Ctrl) Tamoxifen (TAM for 4 days started at day −3, 0, or 5). **b** Kinetics of *Raptor* deletion efficiency days before (day 0) and after starting the 4 days of Tamoxifen (TAM) treatment (day 1, 3, 6, 8, 10, and 12) (*n* = 4). **c** AML burden with or without inducible *Raptor* deletion in combination with chemotherapy (*n* = 8). **d** Experimental design of retroviral transduction of *hRAPTOR* for rescue of *Raptor* deletion: The number of AML cells was evaluated at day 10 after initiation of chemotherapy (Dox + AraC). **e** AML burden with or without rescue: AML cell number per femur was evaluated. Empty vector and *hRAPTOR* were transfected (empty vector: *n* = 8, *hRAPTOR* *n* = 10). **b**–**c**, **e** Representative data from two independent experiments were shown. Mean ± SEM was shown in bar pot (each dot represents each sample). Statistical analysis was performed by using two-way *t*-test (**e**) and paired *t*-test (**b**, **c**) (*p < 0.05, **p < 0.01, ***p < 0.001).

Although mTORC1 inhibitors repress mTORC1 activity and the proliferation of AML cells in vitro (Supplementary Fig. 6a–c), no clear therapeutic effect of mTORC1 inhibition has been observed in clinical trials^8,12,37^. However, these mTORC1 inhibitors were mainly used as monotherapy for established AML, settings our data would suggest are likely to be mTORC1 low and therefore likely unaffected. We hypothesized that timed mTORC1 inhibition might change its anticancer efficacy. We tested this hypothesis using inducible deletion of *Raptor*, a critical component of mTORC1^38^. A primary MLL-AF9 AML cell line derived from *Raptor*^fl/fl^ mice was established and retrovirally transduced with CreERT2. The Cre recombinase was induced in animals with established leukemia by tamoxifen injection at different time points in relation to chemotherapy (Fig. 5a). The induction of *Raptor* deletion at times that overlapped with chemotherapy (day −3 or 0) demonstrated marked increased killing of AML cells compared with later Cre induction (Fig. 5b, c), though the induction process is not immediate, nor is it absolutely efficient (Fig. 5b). Notably, if we transduced *hRAPTOR* into the *Raptor* deleted AML cells, the AML survival was rescued (Fig. 5d, e). The effect of inhibiting mTORC1 on AML was greatest if *Raptor* was deleted coincident with the expected induction in mTORC1 activity after chemotherapy. These results indicate that mTORC1 inhibition improved chemotherapy efficacy if timed inhibition during the peri-chemotherapy period was effectively achieved.

## Discussion

While mTORC1 inhibitors were found to repress AML cell proliferation in vitro^7^, the efficacy of mTORC1 inhibitors in vivo is limited^8,37^. To explain this discrepancy, several mechanisms have been suggested such as limited functional inhibition of mTORC1 in vivo, activation of alternative pathways achieving mTORC1 signaling or limited regional delivery^7^. However, these mechanisms do not consider the possibility that mTORC1 might be dynamically regulated in a context-dependent manner in vivo, or that mTORC1 might be already suppressed in the context of a “packed” leukemic marrow. Since mTORC1 activity can be affected by microenvironmental components such as the availability of nutrients and cytokines^3,5–8^, we sought to study the dynamics of mTORC1 activity in vivo. To this end, we developed an mTORC1 biosensor, mVenus-TOSI. This probe can detect changes of mTORC1 activity induced by serum, growth factors or administration of mTOR inhibitors in an inversely correlated manner with a 3 to 4-fold dynamic range, which is higher than that of a previously reported FRET-based mTORC1 probe (1.16-fold dynamic range)^39^.

Using this probe, we found mTORC1-high and low populations in an AML model with functional and gene expression correlates of those populations. In addition, in vivo imaging and flow cytometric analysis revealed that there was intra-tumoral heterogeneity of mTORC1 activity. Imaging data also implied that mTORC1 activity might depend on the location in the bone marrow. We further found that mTORC1 activity was negatively correlated with AML burden, perhaps due to the limited availability of key nutrients. Similarly, there is heterogeneity in mTORC1 activity among human AML cases as shown from analysis of microarray data and from early phase clinical trials^40^. Interestingly, our analysis showed that AML cases with mTORC1 high activity correlated with a poor prognosis. Chemotherapy in vitro selectively killed mTORC1 high cells without activating mTORC1 signaling likely due to the persistence of the cell cycle dependent killing agents in the culture continuing to eliminate mTORC1 high cells. On the other hand, chemotherapy treatment in vivo is followed by clearance of AML and by the secondary increased availability of nutrients and/or oxygen as the leukemic burden decreases^41,42^. Our barcoding experiments indicate that this is due to induction of mTORC1 activity post chemotherapy rather than selection of mTORC1 high cells. In aggregate, these data point to the value of dynamic measurement of pathways in vivo to understand changing activation and therefore, vulnerabilities to inhibitors.

The shift in mTORC1 activity after chemotherapy suggests that mTORC1 inhibition has the greatest potential for AML cell killing at particular time points. By applying timed genetic deletion of *Raptor*, an essential component of mTORC1, we could observe improved killing of AML cells when *Raptor* was deleted with chemotherapy. While a very different set of circumstances apply, it is interesting to note that a recent clinical trial used a Rapalog, Everolimus, in combination with chemotherapy in childhood acute lymphoid leukemia and observed a favorable outcome^43^, though there were failures of mTORC1 inhibitors used as monotherapy. Similarly, a single-arm study showed that the combination of the mTORC1 inhibitor, Sirolimus with chemotherapy in adult high-risk AML patients could be beneficial for AML with higher mTORC1 activity. Sirolimus administration was started 3 days earlier than chemotherapy, a time we found to be suboptimal^40^. The combination of mTORC1 inhibition at specific times following specific types of chemotherapy may be worth testing in AML.

Further analysis is needed to reveal the contributions of the microenvironment to mTORC1 activity during AML progression/regression and appropriate timing and inhibitor combinations of mTORC1 inhibition for AML treatment. Nonetheless, we propose mVenus-TOSI as a useful biosensor for dynamic imaging of mTORC1 activity that reveals mTORC1 signaling to be dynamic in vivo depending on the state of disease or treatment. The dynamic monitoring of molecular functions in AML and perhaps other cancers may enable more effective combinations of agents in a timed manner to improve disease outcomes.

## Methods

### Plasmids

Full length or partial fragments (PDCD4, PDCD4 (1–80), PDCD4 (1–100), PDCD4 (62-76; Deg)) of mouse PDCD4 were amplified from mouse spleen-derived cDNA using primers with 5′-XhoI and 3′-Not I site by PCR. These fragments were subcloned into a pMXs-internal ribosome entry site (IRES)-Puro vector (pMXs-IP). The fragments for mVenus or mVenus with SV40NLS (mVenusNLS) were amplified from pMXs-IP-mVenus-p27K^−^^22^ using primers with 5′-EcoRI and 3′-XhoI site by PCR and subcloned into pMXs-IP with PDCD4 fragments. MSCV-IRES-KO and MSCV-IRES-KO-hRPTOR plasmids were kind gifts from Dr. Takayuki Hoshii^6^. The 5′half of SCAT3.2 probe was generated by amplifying Amcyan using primers with 5′-BamHI and 3′-EcoRI sites from pMXs-Amcyan-hGEMININ^22^ and pCDNA3-SCAT3^34^. The 3′ half of SCAT3.2 were generated by cleaved from pCDNA3-SCAT3 with EcoRI and NotI. Both 5′ and 3′ half of SCAT3.2 were subcloned into pMX-IP.

### Mouse AML cell lines

Mouse granulocyte macrophage progenitor (GMP)-derived pre-leukemic cells were generated with retroviral transduction of the MLL-AF9 oncogene as described previously^18^. These cell lines were transplanted into mice and AML cells were harvested after the mice developed AML. AML cells with mVenus-TOSI (FM4-mVenus or FM4-mVenus-TdT) were generated by retrovirally transducing with pMXs-IP-mVenus-TOSI and pLenti-iRFP with or without pMXs-IRES-TdTomato and were selected with drug and FACS sorting followed by picking single cell derived clones. Similarly, FM4-SCAT3.2 and FM4-mVenus-p27K^−^ were developed. Other mouse pre-leukemic cell lines were generated similarly from mouse GMP (CL3) or hematopoietic stem cells (KLS-1). For generation of the *Raptor* KO mouse model, AML cells (*Raptor*^*fl/fl*^ AML) were similarly generated by transducing MLL-AF9 to GMP cells derived from *Raptor*^*fl/fl*^ mice and transplanted into recipient mice. Then CreERT2 was retrovirally introduced into *Raptor*^*fl/fl*^ AML cells with MSCV-IRES-CreERT2 (CreERT2 *Raptor*^f*l/fl*^ AML). A mouse AML cell line with MLL-Septin6 (HF6) were a kind gift from Dr. Toshio Kitamura^44^.

### Reagents

Anti-mouse S6K, S6, 4EBP, PDCD4, beta-Actin, phospho-S6K, phosphor-S6, phosphor-4EBP, and phospho-4EBP-Alexa647 antibodies were purchased from Cell Signaling Technologies (Danvers, MA, USA). Anti-mouse phoshoS6-PE, anti-mouse CD45.1 Pacific Blue, and anti-mouse Ki67 Brilliant Violet 605 antibodies were purchased from Biolegend (San Diego, CA, USA). Anti-GFP antibody purchased from Abcam (Cambridge, UK). Cytarabine arabinoside (Ara-C) and doxorubicin were purchased form Sigma-Aldrich (St. Louis, MO, USA). Rapamycin, Rad001, Torin2, AZD0855, and MLN(0128) were purchased from Selleckchem (Houston, TX, USA).

### Cell culture

NIH 3T3 cells were cultured in DMEM supplemented with 10% FBS and penicillin/streptomycin (DMEM/10%FBS). Mouse AML cell lines were cultured in RPMI1640 supplemented with 10% FBS and 3 ng/ml IL-3, 10 ng/ml SCF and penicillin/streptomycin (the AML culture medium). For proliferation assays, relative cell numbers before and after culture were analyzed with CellTiter-Glo (Promega, Madison, WI, USA). For colony formation assays, 1000 AML cells were suspended in 1 ml MethoCultTM GF M3434 (StemCell Techonologies, Vancouver, Canada), plated in 3.5 cm dishes and counted after 7 days.

### Mouse AML model

Mouse AML was developed by transplanting mouse AML cell line into sublethally irradiated (4.5 Gy) mice as described^18^. For rapamycin treatment, the transplanted mice were treated with intraperitoneal injection (i.p.) of 4 mg/kg rapamycin or vehicle as described^6^. For chemotherapy treatment, the transplanted mice were treated with i.p. of cytosine arabinoside 100 mg/kg for 5 days and doxorubicin 3 mg/kg for 3 days concurrently as described^18^ and the treatment was started 21 days after bone marrow transplantation. I.p. injection of 10 mg/kg tamoxifen or vehicle for 4 days was started at day −3, 0, or 5 of chemotherapy in order to delete *Raptor*. For flow cytometry analysis, AML cells were harvested from bone marrow by bone marrow aspiration or sacrificing animals. The animal study protocol was approved by Massachusetts General Hospital Institutional Animal Care and Use Committee (IACUC).

### Flow cytometry

Flow Cytometry analyses were conducted with BD FACS Aria II, BD LSRII, and BD FACS caliber (Becton Dickinson (Franklin Lakes, NJ, USA)) following manufactures protocols. For mTOR signal detection, mVenus with/without iRFP signal intensity were measured by flow cytometer. mTORC1 low (mVenus high) cells was defined by the cells with mVenus intensity higher than lower 10th percentile of mVenus intensity of the reference cells (FM4-venus cells which was independently treated with 100 nM rapamycin for 24 h). For cell cycle analysis, G0 cell were detected by intensity of mVenus signal from FM4-mVenus-p27K^−^ or negative Ki67 staining as reported^22^. For apoptosis cell detection, FRET and Amcyan signal from FM4-SCAT3.2 were detected similarly as described previously^34^.

### Intravital imaging

Intravital imaging of mouse calvarial bone marrow was performed with a custom made 2-photon or confocal microscope, or Olympus 2-photon microscope system (Olympus (Tokyo, Japan)) as described^41,45^. At least 2 mice were imaged for each condition and representative data shown. Each dot represents an individual cell. For analysis of the distance from bone, mTORC1 activity low (mVenus high = yellow) cells were determined by the ratio between signal intensity of mVenus/TdTomato (channel 2/channel 1) >=0.4 and mTORC1 high (mVenus low = red) were defined by the ratio <0.4. The distance between the bone surface and the center of the individual cell was determined based on the z-stack images as described^41,46^. Briefly, 3D models of these cells and the bone (second harmonic generation) were developed through z-stack reconstruction. In Supplementary Fig. 2e, the shortest distance for to the endosteum was determined in three dimensions using the Pythagorean Theorem. The data are pooled from two mice and both of individual mice showed same tendency. In Fig. 4c, in order to show entire calvarial bone marrow, we stitched images of 48 visual fields and the maximum intensity z-projection image displayed in the red and green channel. In order to show change in mVenus intensity in AML cells with probe, the bone signal (blue channel) was not included in the z-projection.

For survival and apoptosis imaging, FRET (mVenus) signal (channel 2) and Amcyan signal (channel 1) were collected and the ratio between mVenus/Amcyan was calculated in order to normalize FRET signal. The images were analyzed using Olympus FV10-SW software and/or Image J software (http://rsbweb.nih.gov/ij/) and further analysis was done with R (https://www.r-project.org) or Matlab (MathWorks, Natick, MA, USA).

### RNA sequencing and analysis

Fifty thousand mVenus-high and mVenus-low AML cells with mVenus-TOSI (FM4-Venus) were sorted from bone marrow cells of each of 3–4 mice with AML treated with vehicle or chemotherapy regimen at day 7 (3 days after the end of chemotherapy). RNAs were extracted from samples with a DNA/RNA micro kit (Qiagen (Hilden, Germany)), used for making a sequencing library with Illumina Truseq kit, and subjected to sequencing with Next-seq (core at Boston University). RNAseq data was analyzed with STAR^47^ for alignment and counting the reads, then the data was preprocessed and analyzed with EdgeR^48^. The complete gene data set was analyzed using GenePattern (http://software.broadinstitute.org/cancer/software/genepattern/) for gene set enrichment analysis (GSEA) as described^49^. Gene Signatures used here were collected from MSigDB (http://software.broadinstitute.org/gsea/msigdb/index.jsp) or publications^20–22,24,25^ and gene-set overlap analysis was conducted with the Investigate Gene Sets fuction of MSigDB.

### Human AML microarray and single cell RNA-seq data analysis

The microarray data for human AML patients (GSE12417)^28^ were used for clustering and survival analysis with R. The *p*-values for the difference between mTORC1 high and low group at different time points were also automatically calculated with logrank test. Differentially expressed genes were analyzed with genefilter package (https://bioconductor.org/packages/release/bioc/html/genefilter.html). The single cell RNA-seq data for human AML patients (GSE116256)^29^ were used for clustering and sub-population analysis for mTORC1 related pathway analysis with MSigDB. Single cell RNA-seq data analysis was performed with R and Seurat Package (http`s://satijalab.org/seurat/)^50,51^.

### Clonal diversity analysis with the ClonTracer

Two million FM4-Venus cells were subjected to lentiviral infection of the ClonTracer library plasmid^33^ with DsRed selection marker (Addgene (Cambridge, MA, USA)) with MOI < 10%. They were incubated for 3 days and sorted with FACS AriaII (BD). One million sorted cells were obtained. The sorted cells were expanded up to 1 billion by culturing for 10 days. One million cells were transplanted into each of the four groups of the four mice. Four hundred thousand mVenus-high or low AML cells were sorted from the mouse bone marrow by sacrificing the mice immediately before (day 0) or at either of the 3 different time after chemotherapy (day 5, 7, and 14). Genomic DNA was extracted with the DNA/RNA mini kit (Qiagen) and sequencing libraries were constructed according to the protocol from Addgene (https://www.addgene.org/pooled-library/clontracer/). The resulting sequencing libraries were pooled and subjected with Illumina High-seq to DFCI sequencing core. The data was analyzed with the Python program provided by Addgene for alignment (https://www.addgene.org/static/cms/filer_public/85/8a/858a70b5-bc3c-4598-8af1-26c3b6419006/clontracer_analyze_12.zip) and further analysis was conducted by R.

### Statistical analysis

Statistical significance was calculated using the Student, Welch *t* test or Analysis of Variance (ANOVA) and paired *t* test for independent variables. ANOVA was used for initial analysis for Figs. 1e, 1j, 2d, 2g, 2h, 4b, 4d–f, 5b, 5c and subsequent subclass analysis was performed with paired *t* test. Survival was analyzed with log rank test.

### Reporting summary

Further information on research design is available in the Nature Research Reporting Summary linked to this article.

## Supplementary information

Supplementary Information

Description of Additional Supplementary Files

Supplementary Movie 1

Supplementary Movie 2

Supplementary Movie 3

Reporting Summary

## Data Availability

The RNA-seq data have been deposited in the GEO database under the accession code GSE161362 (Fig. 3) and GSE161490 (Fig. 4). The DNA sequencing data for the ClonTracer barcode have been deposited in the GEO database under the accession code GSE162153 (Fig. 4 and Supplementary Fig. 4). The microarray data for human AML patients (GSE12417)^28^ for Fig. 3 and Supplementary Fig. 3 and the single cell RNA-seq data for human AML patients (GSE116256)^29^ for Supplementary Fig. 3 are publicly available in the GEO database. All the other data supporting the findings of this study are available within the article and its supplementary information files and from the corresponding author upon reasonable request. Source data are provided with this paper.

## Source data

Source Data

## Code availability

Data analysis for RNA-seq was conducted by R and Phython other than STAR for alignment and counting the reads, as well as GenePattern and MSigDB for gene set enrichment analysis. Image data analysis was done by Image J, Matlab, and R.The custom codes or algorithms will be available at Github (https://github.com/maromato/Imaging_dynamic_mTOR) or will be available upon request.
