# Supplementary material for: Imaging dynamic mTORC1 pathway activity in vivo reveals marked shifts that support time-specific inhibitor therapy in AML
Source: Nat Commun. 2021 Jan 11;12:245. doi: 10.1038/s41467-020-20491-8 (PMC7801403; doi:10.1038/s41467-020-20491-8)
Supplement: Supplementary file 1 — Supplementary Information [file 41467_2020_20491_MOESM1_ESM.pdf]

**Supplementary information**

**for**

# Imaging dynamic mTORC1 pathway activity in vivo reveals marked shifts that support time-specific inhibitor therapy in AML

Toshihiko Oki [1,2,3], Francois Mercier [1,2,3,7,*], Hiroki Kato[1,2,3,*], Yookyung Jung [4], Thomas O. McDonald [2,5,9], Joel A. Spencer [3,6], Michael C. Mazzola[1,2,3], Nick van Gastel[1,2,3], Charles P. Lin [3,4], Franziska Michor [2,5,9,10,11], Toshio Kitamura[8] and David T. Scadden [1,2,3,11]

**Affiliations**

1. Center for Regenerative Medicine, Massachusetts General Hospital, Boston, MA, 02114, USA
2. Department of Stem Cell and Regenerative Biology, Harvard University, Cambridge, MA 02138
3. Harvard Stem Cell Institute, Harvard University. Cambridge, MA, 02138
4. Wellman Center for Photomedicine, Massachusetts General Hospital, Boston, MA, 02114, USA
5. Center for Cancer Evolution and Department of Data Sciences, Dana-Farber Cancer Institute, Boston, MA, 02215, USA
6. Present address: School of Engineering, University of California, Merced, Merced, CA, 95343, USA
7. Present address: McGill University, Montreal, Canada
8. Division of Cellular Therapy, The Institute of Medical Science, University of Tokyo, 108-8639, Japan
9. Department of Biostatistics, Harvard T.H. Chan School of Public Health, Boston, MA, 02215, USA
10. The Broad Institute of Harvard and MIT, Cambridge, MA 02139, USA
11. The Ludwig Center at Harvard, Boston, MA 02215, USA

* These authors contribute equally.

Supplementary figure 1

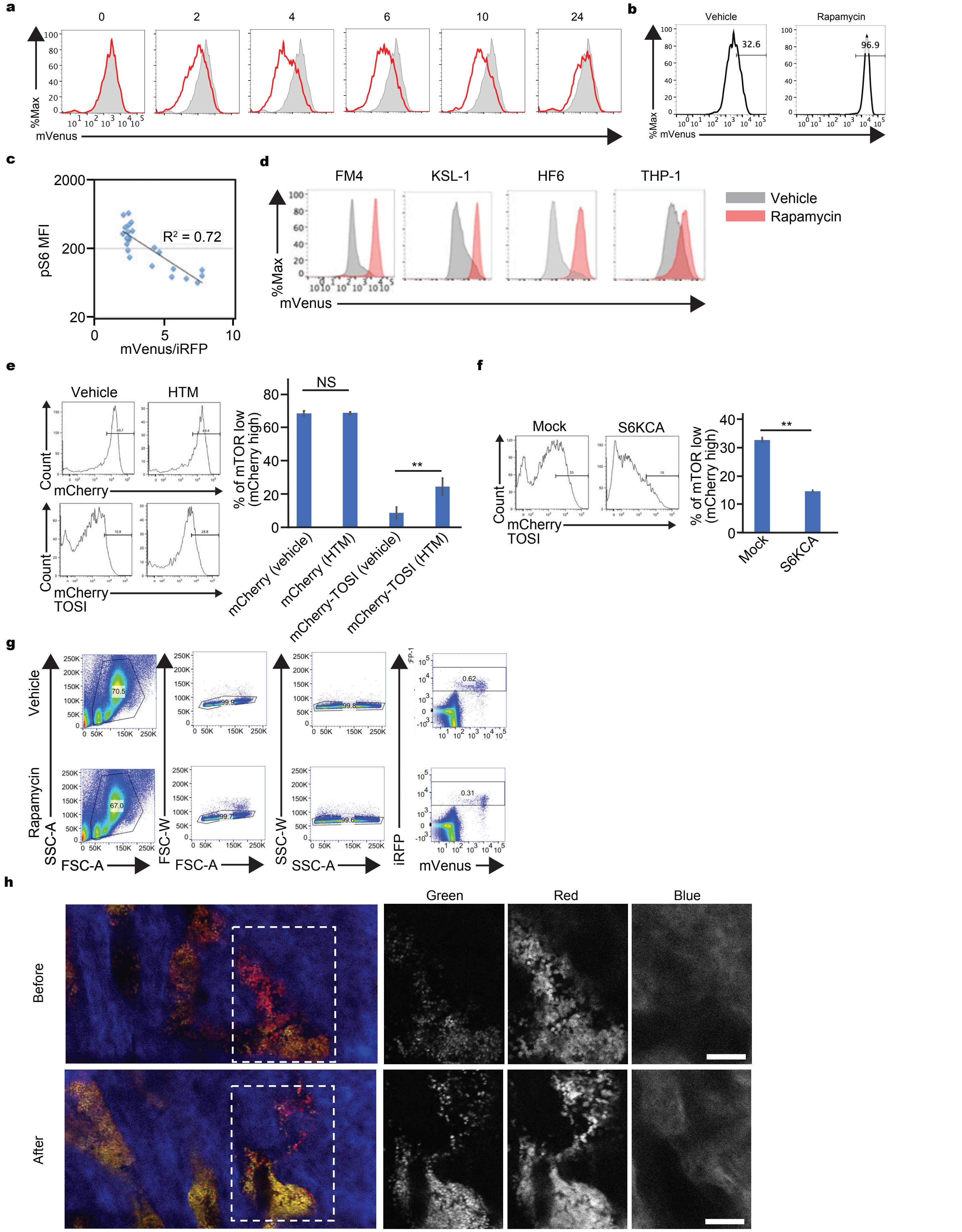


Supplementary figure 1 Characteristics of PDCD4 probes

(a) Representative histogram plots in NIH 3T3 at indicated time (hour) after serum refeeding (red) comparing to that of 0 hour (gray). (b) Representative histogram plots for the detection of mVenus intensity in AML cells (FM4) in response to vehicle (left) and rapamycin (right). (c) Correlation of pS6 and mVenus/iRFP (1/mTORC1 activity) in AML cells (FM4) in vitro. FM4 cells were stimulated with IL-3/SCF and different concentration of Torin 2 (0, 0.001, 0.01, 0.1, 1, 10, 100, 1000 nM) for 24 hours to produce sets of AML cells with different mTORC1 activity. (d) Representative histogram plot of mVenus intensity changes by rapamycin treatment in indicated mouse (FM4, KSL-1 (KSL-AML), HF6) and human (THP-1) AML cell lines. The bar plots of this experiments were shown in Fig S6a. (e) mCherry intensity changes measured by flow cytometry in CreERT-Raptorfl/fl AML transduced with mCherryNLS or mCherry-TOSI 48 hours after vehicle or 4-hydroxytamoxifen (HTM 0.01 μM). The cells were stimulated with IL-3/SCF for 24 hours. Representative histogram (left) and the bar chart for % of mCherry high population (n = 4) (right). (f) mCherry intensity changes measured by flow cytometry in mouse MLL-AF9 AML (CL3) cells transduced with MSCV-IG (Mock) or MSCV-IG-S6KCA (S6KCA) and mCherry-TOSI. The cells were stimulated with IL-3/SCF for 24 hours. Representative histogram (left) and the bar chart for % of mCherry high population (n = 3) (right). (g) Representative flow cytometry plots for FSC and SSC of mVenus intensities in AML cells harvested from mice treated with vehicle or rapamycin (Supplemental data for Fig1G). (h) (Left) Low magnification image for Figure 1I. Boxed area are used for Figure 1I (Right) Individual channel (Green (mVenus), Red (mCherry), Blue (Bone signal)) of Figure 1i for in vivo imaging with a 2-photon microscope of the calvarial bone marrow before and 24 hours after rapamycin treatment (4 mg/kg i.p.) from a similar visual field of same mouse. Scale bar = 100 μm. (a-h) Representative data from at least two independent experiments were shown. Statistical analysis was performed by using two-way t-test (**p<0.01). Source data are provided as a Source Data file.

Supplementary figure 2

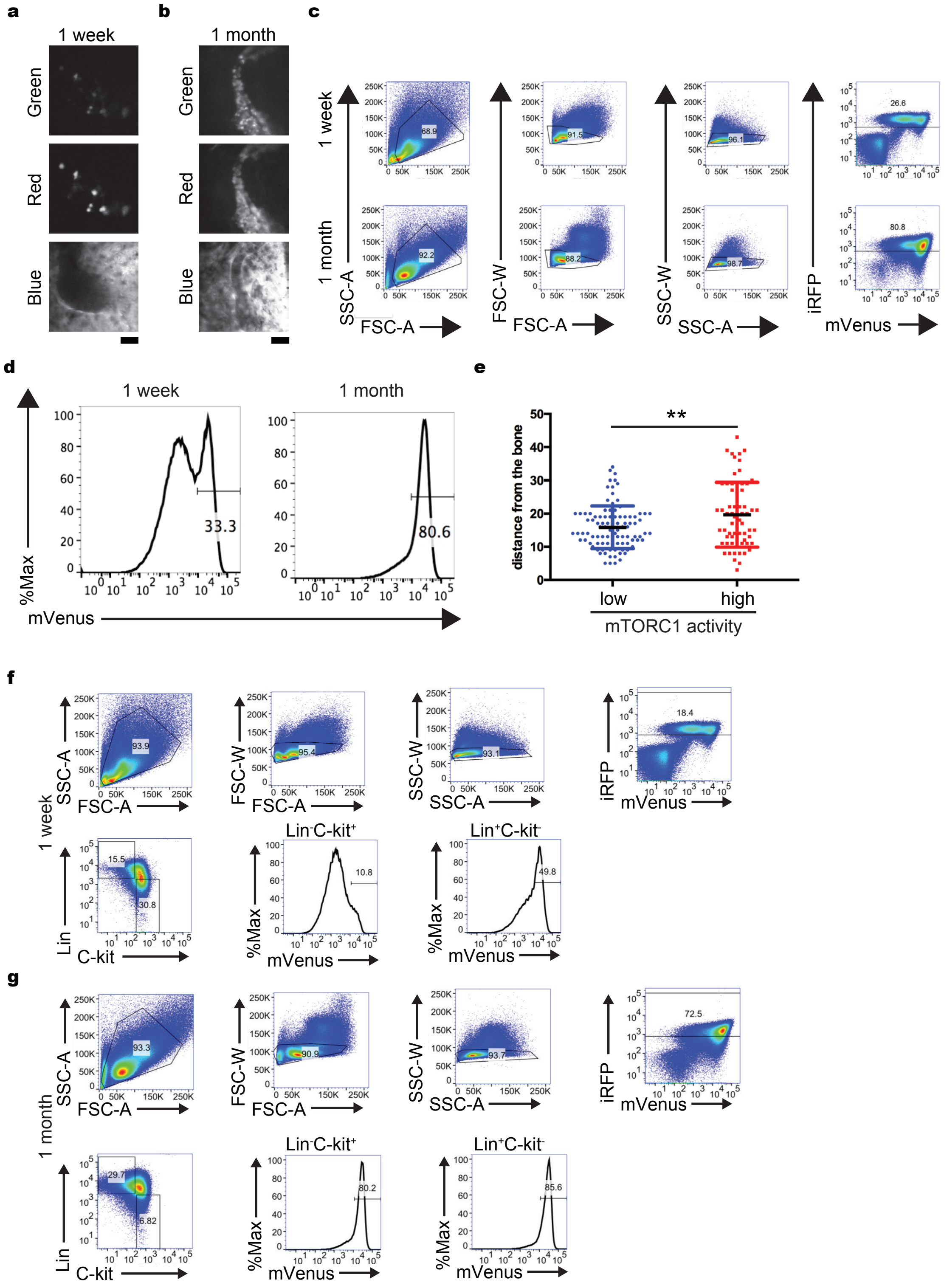


Supplementary figure 2 mTORC1 activity depending on the AML burden and cell localization

(a-b) Representative images for individual channel (Green (mVenus), Red (Tdtomato), and Blue (Bone signal)) of Figure 2a for in vivo imaging of AML cell with mVenus-TOSI probe (FM4- Venus-TdT) in the mouse calvarial bone marrow from similar visual fields of the mouse using a confocal microscope during disease development at 1 week (a) and 1 month (b) after transplantation. Scale = 50 μm.

(c-d) Representative flow cytometry plot of mVenus-TOSI probe intensity during disease development at 1 week and 1 month after transplantation (FSC and SSC (c) and histogram of mVenus intensity (d)).

(e) The distance (μm) between mTORC1 activity low (mVenus/Tdtomato >= 0.4) or high cells (mVenus/Tdtomato < 0.4) and the bone surface at 1 week after transplantation (n = 2).

(f-g) Representative flow cytometry plot of mVenus-TOSI probe intensity for differentiated (ckit- /lin+) and undifferentiated (ckit+/lin-) cells during disease development at 1 week (f) and 1 month (g) after transplantation.

(a-h) Representative data from at least two independent experiments were shown. Statistical analysis was performed by using two-way t-test (**p<0.01). Source data are provided as a Source Data file.

Supplementary figure 3

**a**

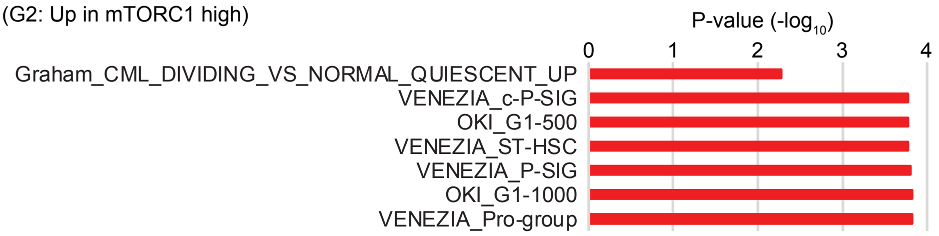


**b**

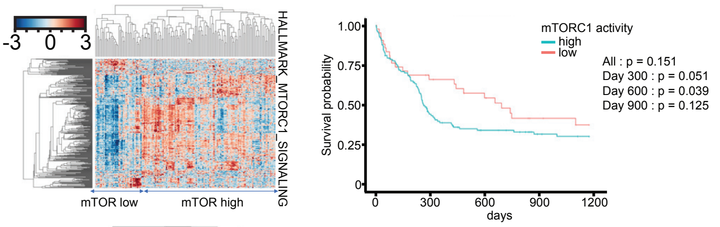


**c**

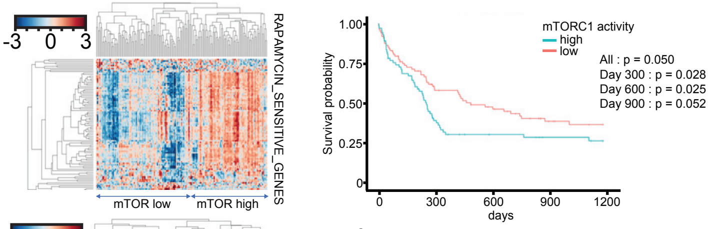


**d**

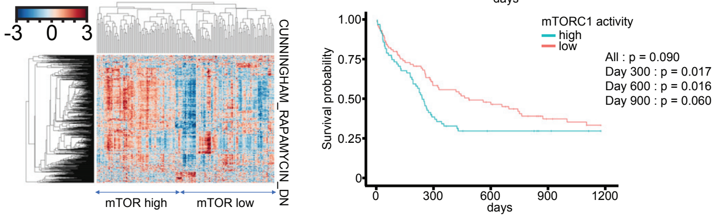


**e**

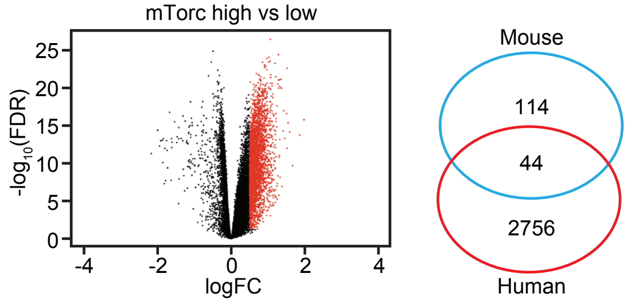


| Gene Set Name | p-value |
|---|---|
| HALLMARK_MYC_TARGETS_V1 | 3.22E-13 |
| HALLMARK_E2F_TARGETS | 1.43E-07 |
| HALLMARK_MYC_TARGETS_V2 | 6.47E-06 |
| HALLMARK_MTORC1_SIGNALING | 2.60E-04 |
| HALLMARK_REACTIVE_OXYGEN_SPECIES_PATHWAY | 4.32E-04 |
| HALLMARK_ALLOGRAFT_REJECTION | 6.91E-03 |
| HALLMARK_HYPOXIA | 6.91E-03 |
| HALLMARK_P53_PATHWAY | 6.91E-03 |

**f**

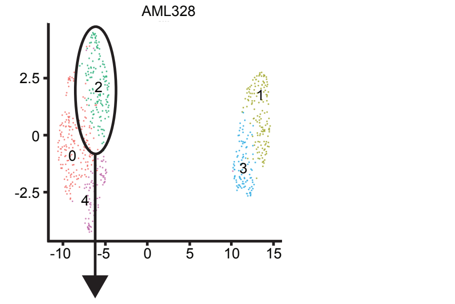


| Gene Set Name | p-value |
|---|---|
| HALLMARK_MYC_TARGETS_V1 | 3.96E-48 |
| HALLMARK_E2F_TARGETS | 3.62E-44 |
| HALLMARK_G2M_CHECKPOINT | 1.20E-17 |
| HALLMARK_MYC_TARGETS_V2 | 4.05E-12 |
| HALLMARK_MTORC1_SIGNALING | 4.56E-09 |
| HALLMARK_GLYCOLYSIS | 8.46E-08 |
| HALLMARK_DNA_REPAIR | 5.98E-05 |
| HALLMARK_FATTY_ACID_METABOLISM | 7.65E-05 |
| HALLMARK_ALLOGRAFT_REJECTION | 2.30E-04 |
| HALLMARK_OXIDATIVE_PHOSPHORYLATION | 2.30E-04 |

**g**

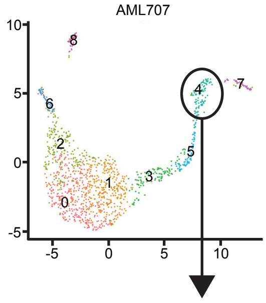


| Gene Set Name | p-value |
|---|---|
| HALLMARK_E2F_TARGETS | 3.86E-56 |
| HALLMARK_G2M_CHECKPOINT | 1.56E-40 |
| HALLMARK_MYC_TARGETS_V1 | 2.22E-15 |
| HALLMARK_MITOTIC_SPINDLE | 5.66E-14 |
| HALLMARK_MTORC1_SIGNALING | 1.29E-08 |
| HALLMARK_DNA_REPAIR | 7.40E-06 |
| HALLMARK_GLYCOLYSIS | 3.97E-04 |
| HALLMARK_CHOLESTEROL_HOMEOSTASIS | 1.57E-03 |
| HALLMARK_APOPTOSIS | 1.58E-03 |
| HALLMARK_ESTROGEN_RESPONSE_LATE | 3.46E-03 |

Supplementary figure 3 Expression profiling between mTORC1 high and low AML cells
(a) Gene set enrichment analysis (GSEA) of differentially expressed genes obtained from mTORC1 high or low AML cells harvested from mouse bone marrow with cell cycle related signatures.(b) Classification of human AML samples (GSE12417) with mTORC1 signal related gene signature (HALLMARK_MTORC1_SIGNALING) (left) and survival probability (right).(c) Classification of human AML samples (GSE12417) with mTORC1 signal related gene signature (BILANGES_RAPAMYCIN_SENSITIVE_GENES) and survival probability (right). (d) Classification of human AML samples (GSE12417) with mTORC1 signal related gene signature (CUNNINGHAM_RAPAMYCIN_DN) (left) and survival probability (right). (e) Highly expressed genes in mTORC1 high human AML (human mTORC high gene signature (mTOR-GS)) and characteristics with overlapped genes between human and mouse mTOR-GS (Fig 3A G2). The highly expressed genes (red dots) in mTORC1 high human AML (human mTOR-GS) (left) and the venn diagram between human and mouse mTOR-GS (middle). Pathway analysis (hypergeomeric test) of overlapped 44 genes between human and mouse mTOR-GS with MSigDB (right). (f-g) Analysis of heterogeneity in mTORC1 signaling within AML patients with single RNA-seq analysis of AML328 (f) and AML707 (g) from human AML samples (GSE116256).

Supplementary figure 4

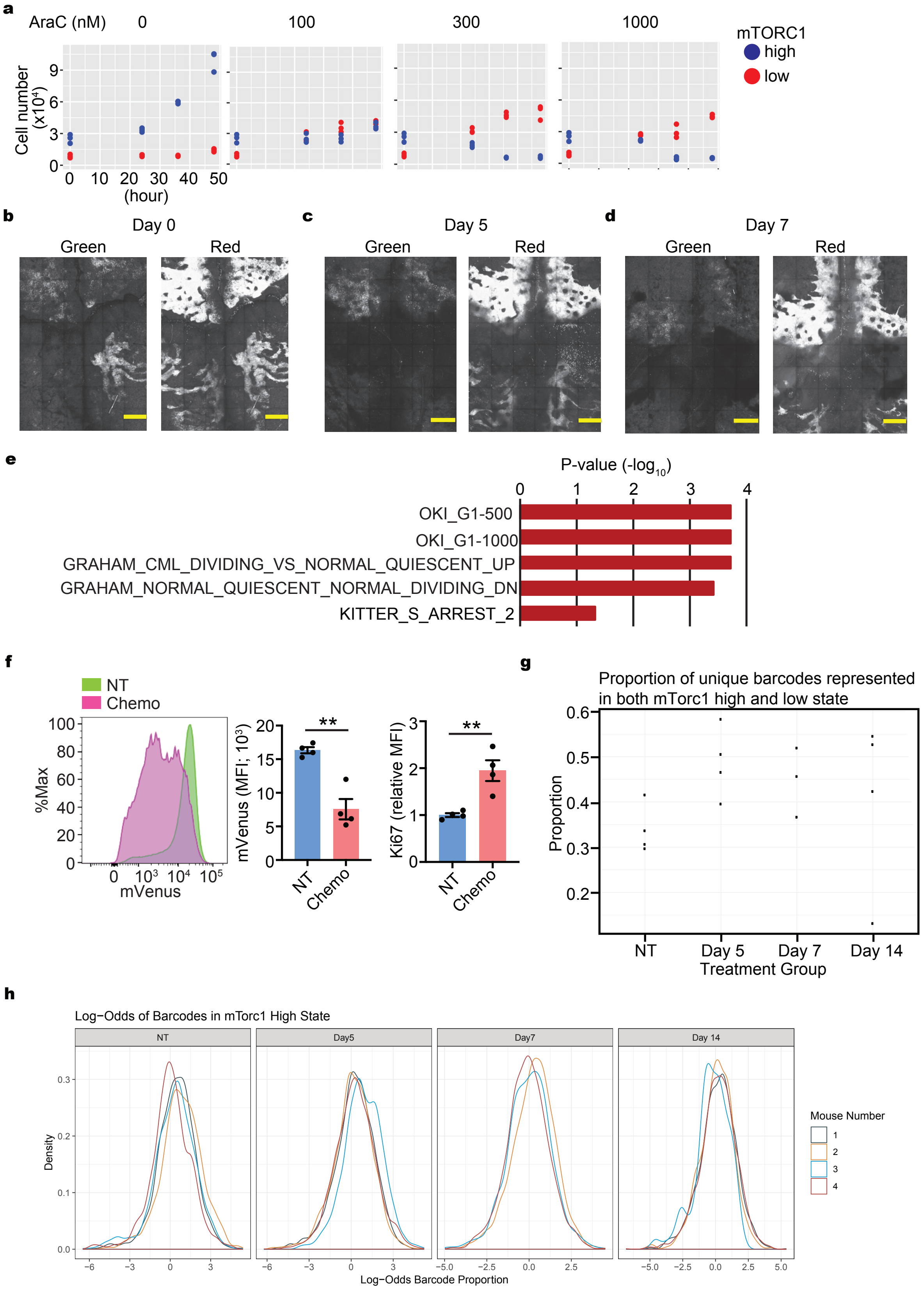


Supplementary figure 4 mTORC1 activity and clonal diversity and apoptosis monitoring during chemotherapy
(a) Cell numbers of mTORC1 high and low cells in vitro with or without Ara-C (0, 100, 300, and 1000 nM) at indicated time (hour) (n = 3).
(b-d) Representative images of individual channel (Green (mVenus) and Red (Tdtomato)) of Fig 4c for intravital imaging of z-projection of the entire calvarial bone marrow with a 2-photon microscope of a FM4-VT transplanted mouse before (day 0) (b) and after chemotherapy (day 5 (c) and 7 (d)), Scale = 500 μm.
(e) Gene set enrichment analysis (GSEA) of highly expressed genes obtained from AML cells from the mice with chemotherapy with cell cycle related signatures.
(f) mVenus intensity and Ki67 intensity of AML cells in vivo with or without chemotherapy. Analysis was performed at 4 weeks after transplantation (n = 4).
(g) Barcode diversity between mTORC1 high and low cells: proportions of the shared barcodes between mTORC1 high and low cells at different time points before (untreated) or after chemotherapy (day 5, 7 and 14) were shown (n = 3 for day 7 and n=4 for NT, day 5 and day 14).
(h) Log-odds ratio of Barcode counts in mTORC1 high state to mTORC1 low state.
(a-d, f) Representative data from two independent experiments were shown. Statistical analysis was performed by using two-way t-test (**$p<0.01$). Source data are provided as a Source Data file.

Supplementary figure 5

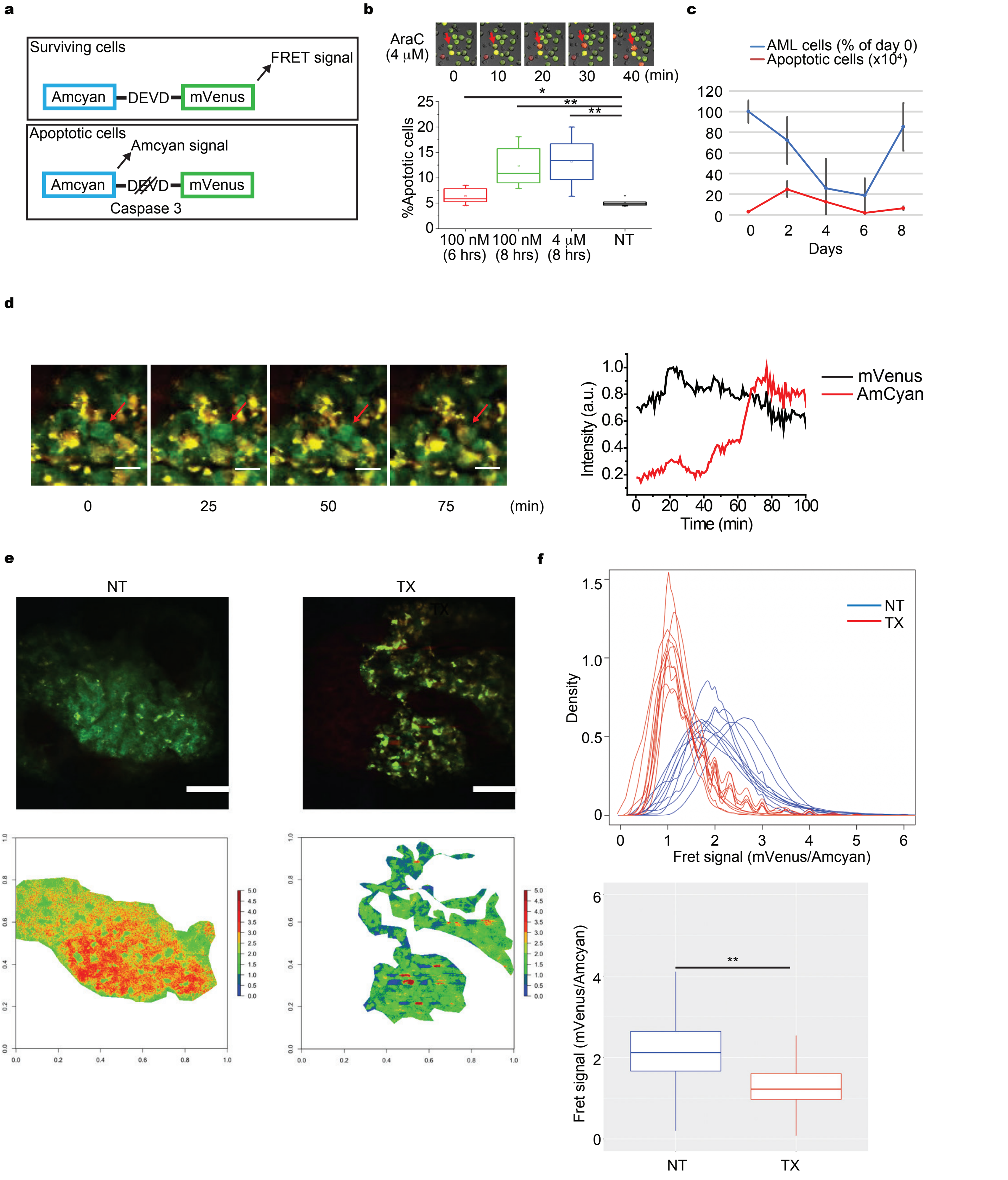


Supplementary figure 5 Visualization of apoptosis process of AML
(a) Schematic design of Scat 3.2 probe.
(b) Visualization of apoptosis process of AML (upper) and proportion of apoptotic AML cells at 6 or 8 hours of Ara-C treatment (lower), Green: FRET positive (live) cells, Red: FRET negative (apoptotic) cells, arrow: cells undergoing apoptosis (n= 11, 10, 15, and 5 for 100 nM (6hr), 100 nM (8hr), 4μM (8hr), and NT, respectively).
(c) Kinetics of numbers of AML cell and apoptotic AML cells before (day 0: the start of chemotherapy) and after chemotherapy (day 2, 4, 6, and 8) in vivo (n =3 for Day 6 and n = 4 for day 0, 2, 4, and 8). Mean SD was shown
in the graph.
(d) Visualization of AML cell apoptosis in vivo with intravital confocal time lapse imaging at day 2 of chemotherapy (left panel) and the time course of Amcyan and FRET signal (mVenus) intensities in an apoptotic AML cell (right panel). Green: FRET positive (live) cells, Yellow: FRET negative (apoptotic) cells, arrow: cells undergoing apoptosis.
(e) The image (upper panel) in one bone marrow cavity from the representative mice with vehicle (NT) (left upper) or chemotherapy (TX) (right upper) at day 6 imaged with intravital confocal microscopy from a visual field. mVenus signal was captured by Green channel and Amcyan were captured by Red channel. Green: FRET positive (surviving cells), Yellow: FRET negative (apoptotic) cells. The ratiometry for normalized FRET signal (mVenus/Amcyan) (lower) of same image was shown.
(f) The summary of the normalized FRET signal (mVenus/Amcyan) from 10 different bone marrow cavities from the representative two mice with vehicle (NT) or chemotherapy (TX) at day 6. Histogram (upper) and box plot (lower).
Mean with interquartile rage was shown in the box pot.
(b-f) Representative data from at least two independent experiments were shown.
Statistical analysis was performed by using two-way t-test (*p<0.05, **p<0.01). Source data are provided as a Source Data file.

Supplementary figure 6

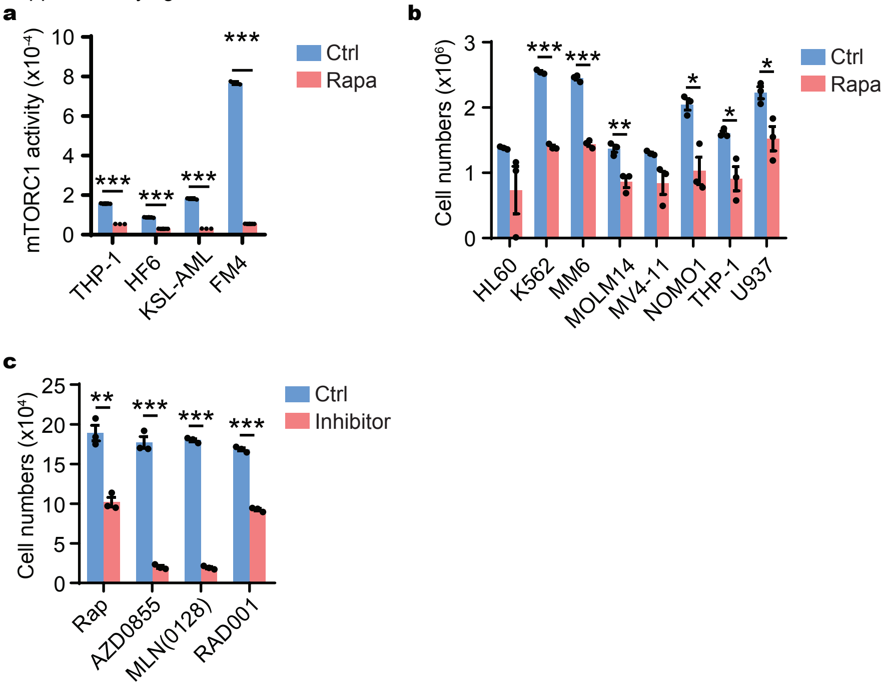


Supplementary figure 6 mTORC1 inhibitor treatment for AML
(a) Effect of rapamycin (100 nM) on mTORC1 activity (1/mVenusMFI) in indicated cell lines (the representative histograms of mVenus intensity were shown in Figure S1d) (n = 3).
(b) Effect of rapamycin (100 nM) on cell numbers in indicated cell lines at day 2 (n = 3). Relative cell numbers were shown.
(c) Effect of indicated mTORC1 inhibitors (100 nM) on FM4 cell numbers at day 2 (n = 3). Relative cell numbers were shown.
(a-c) Representative data from at least two independent experiments were shown.
Mean ± SEM was shown in bar pot (each dot represents each sample). Statistical analysis was performed by using two-way t-test (*p<0.05, **p<0.01, ***p<0.001).
Source data are provided as a Source Data file.
