# Supplementary material for: Imaging dynamic mTORC1 pathway activity in vivo reveals marked shifts that support time-specific inhibitor therapy in AML
Source: Nat Commun. 2021 Jan 11;12:245. doi: 10.1038/s41467-020-20491-8 (PMC7801403; doi:10.1038/s41467-020-20491-8)
Supplement: Supplementary file 2 — Description of Additional Supplementary Files [file 41467_2020_20491_MOESM2_ESM.pdf]

Description of Additional Supplementary Files

Supplementary Movie 1: The time lapse video of apoptosis process of AML for 8 hours of Ara-C treatment (the static images were shown in Supplementary Fig.5b). Green: FRET positive (live) cells, Red: FRET negative (apoptotic) cells.

Supplementary Movie 2: The higher magnification of the time lapse video of AML cell apoptosis *in vivo* with intravital confocal time lapse imaging at day 2 of chemotherapy (the static images were shown in Supplementary Fig. 5d) Green: FRET positive (live) cells, Yellow: FRET negative (apoptotic) cells.

Supplementary Movie 3: The lower magnification of the time lapse video of AML cell apoptosis *in vivo* with intravital confocal time lapse imaging at day 2 of chemotherapy (the static images were shown in Supplementary Fig. 5d) Green: FRET positive (live) cells, Yellow: FRET negative (apoptotic) cells.
