# Supplementary material for: Imaging dynamic mTORC1 pathway activity in vivo reveals marked shifts that support time-specific inhibitor therapy in AML
Source: Nat Commun. 2021 Jan 11;12:245. doi: 10.1038/s41467-020-20491-8 (PMC7801403; doi:10.1038/s41467-020-20491-8)
Supplement: Supplementary file 7 — Source Data [file 41467_2020_20491_MOESM7_ESM.zip › Source data/Source data.docx]

Source data files.

We included the source data in a source data file and revised the figure legend. The source data underlying Fig.1,2,4 and 5 and Supplementary Fig.1,2, and 4-6 are provided as a Source Data file.
