# Supplementary material for: Imaging dynamic mTORC1 pathway activity in vivo reveals marked shifts that support time-specific inhibitor therapy in AML
Source: Nat Commun. 2021 Jan 11;12:245. doi: 10.1038/s41467-020-20491-8 (PMC7801403; doi:10.1038/s41467-020-20491-8)

Western raw data for Fig.1d

- Gel 1 was used for pS6K (long exposure), Venus (mid exposure), pS6 (mid exposure), p4EBP (mid exposure-2).

- Gel 1 re-probe was used for S6K (short exposure), beta-actin (short exposure), S6 (long exposure), 4EBP (mid exposure).

- Gel 2 was used for PDCD4 (short exposure)

- Kaleidoscope (Biorad) was used for protein maker: 250, 150, 100,75, 50,37, 25,20,15 KD were shown in the film.

- The membrane was cut at the level of 50 KD, 37KD, 25KD and each part was blotted with each of antibody.

Gel 1: short exposure

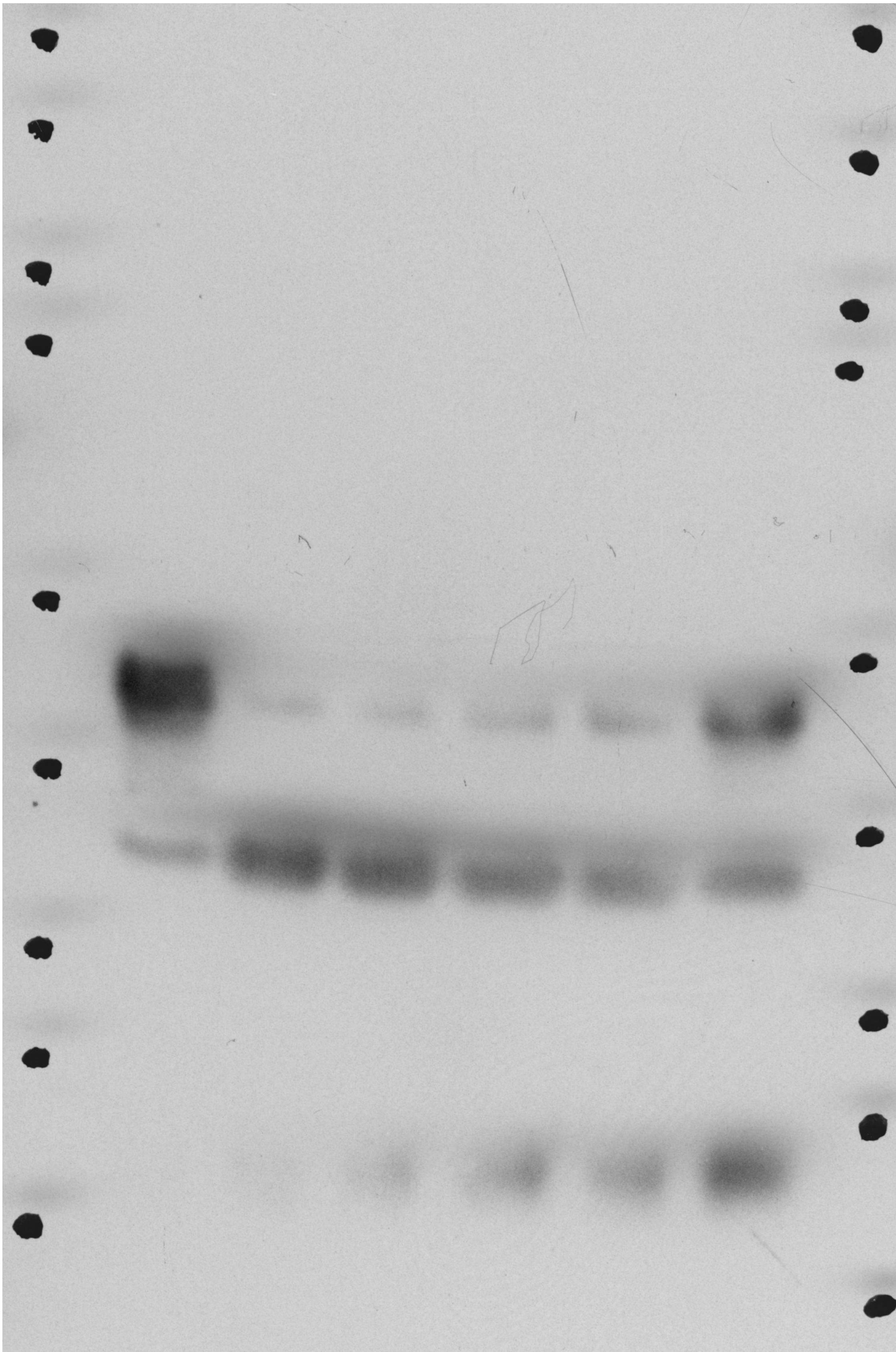

pS6K

Venus

pS6

p4EBP

Gel 1: mid exposure

pS6K

Venus

pS6

p4EBP

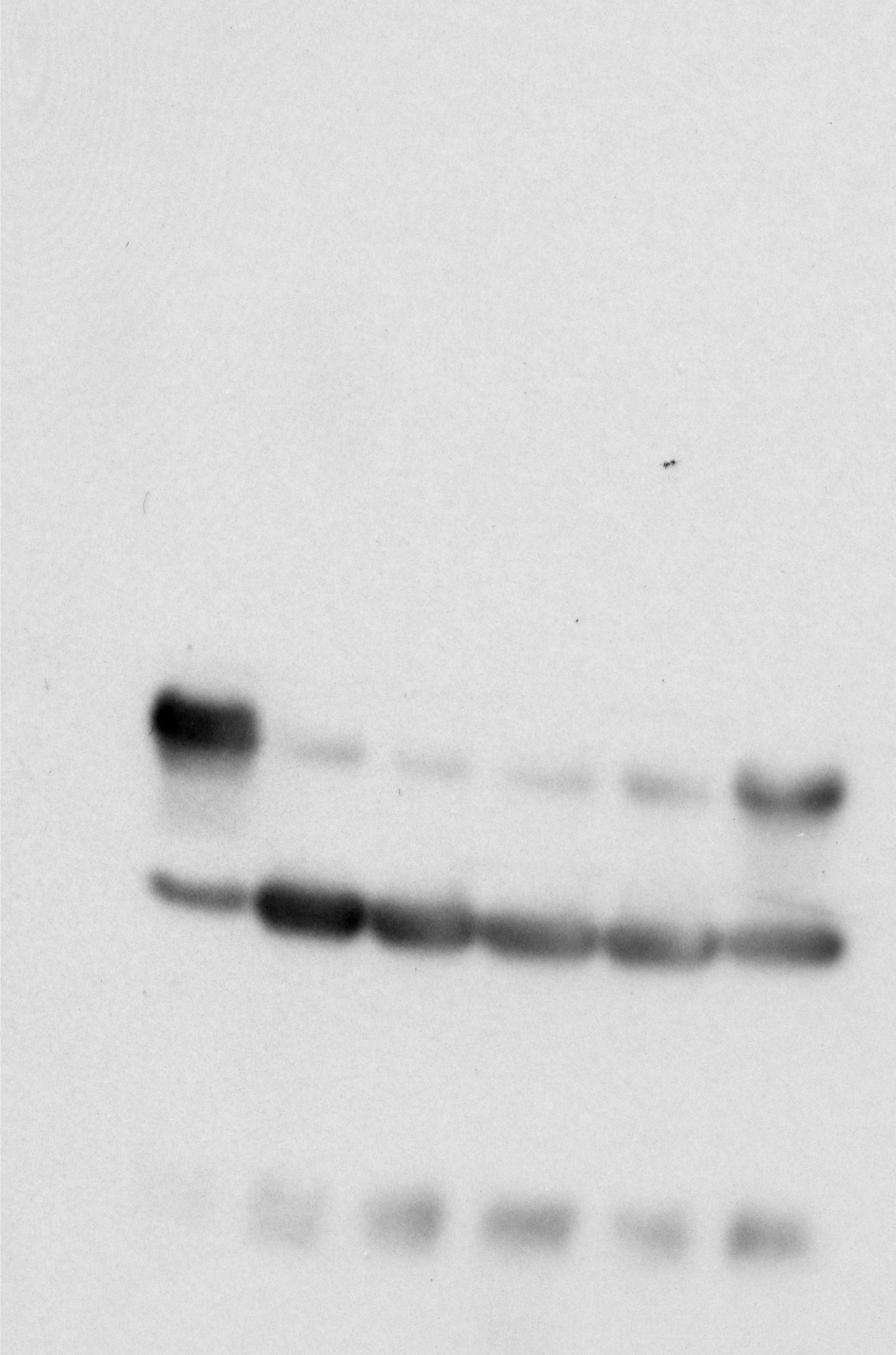

Gel 1: mid exposure-2

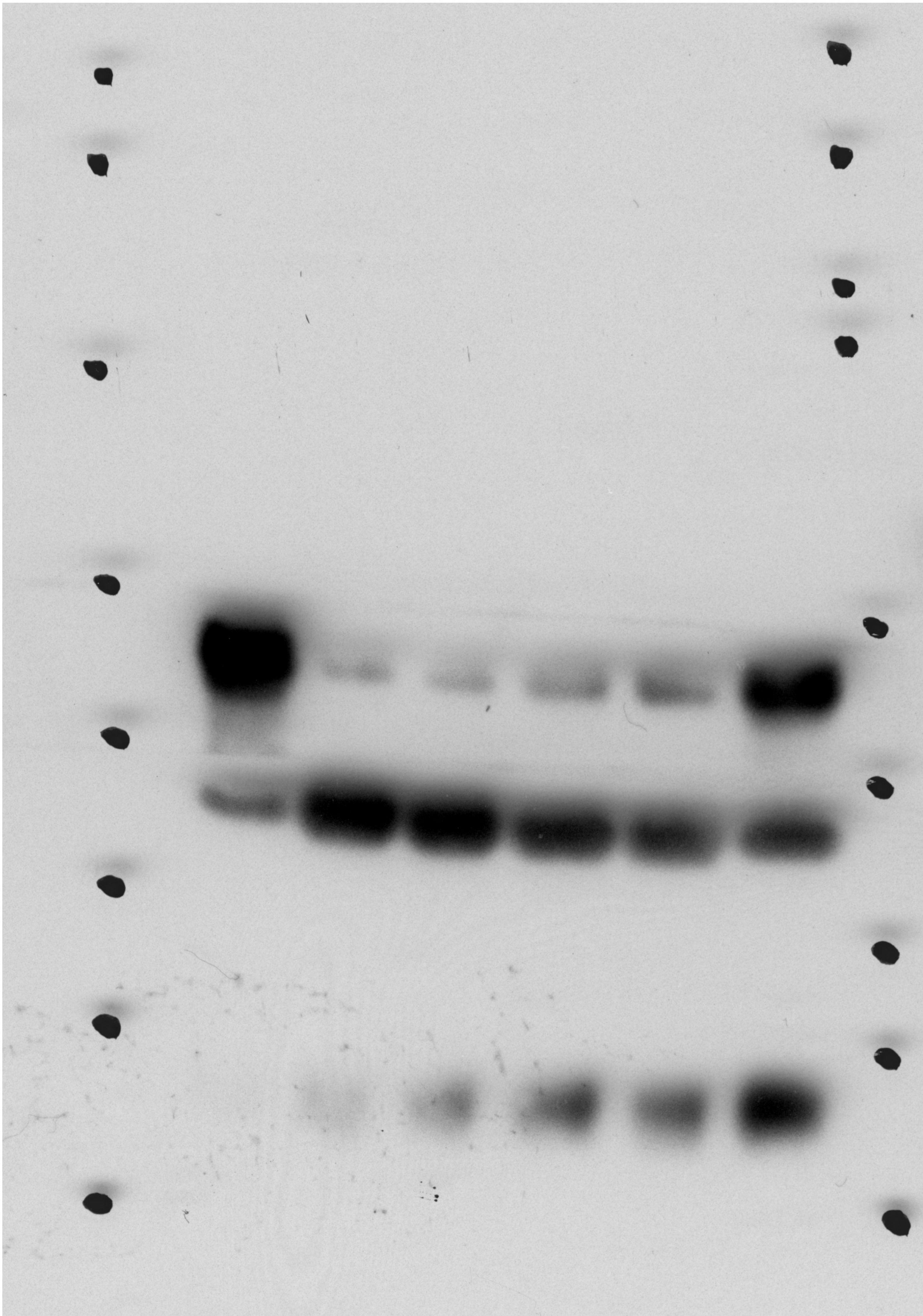

Gel 1: long exposure

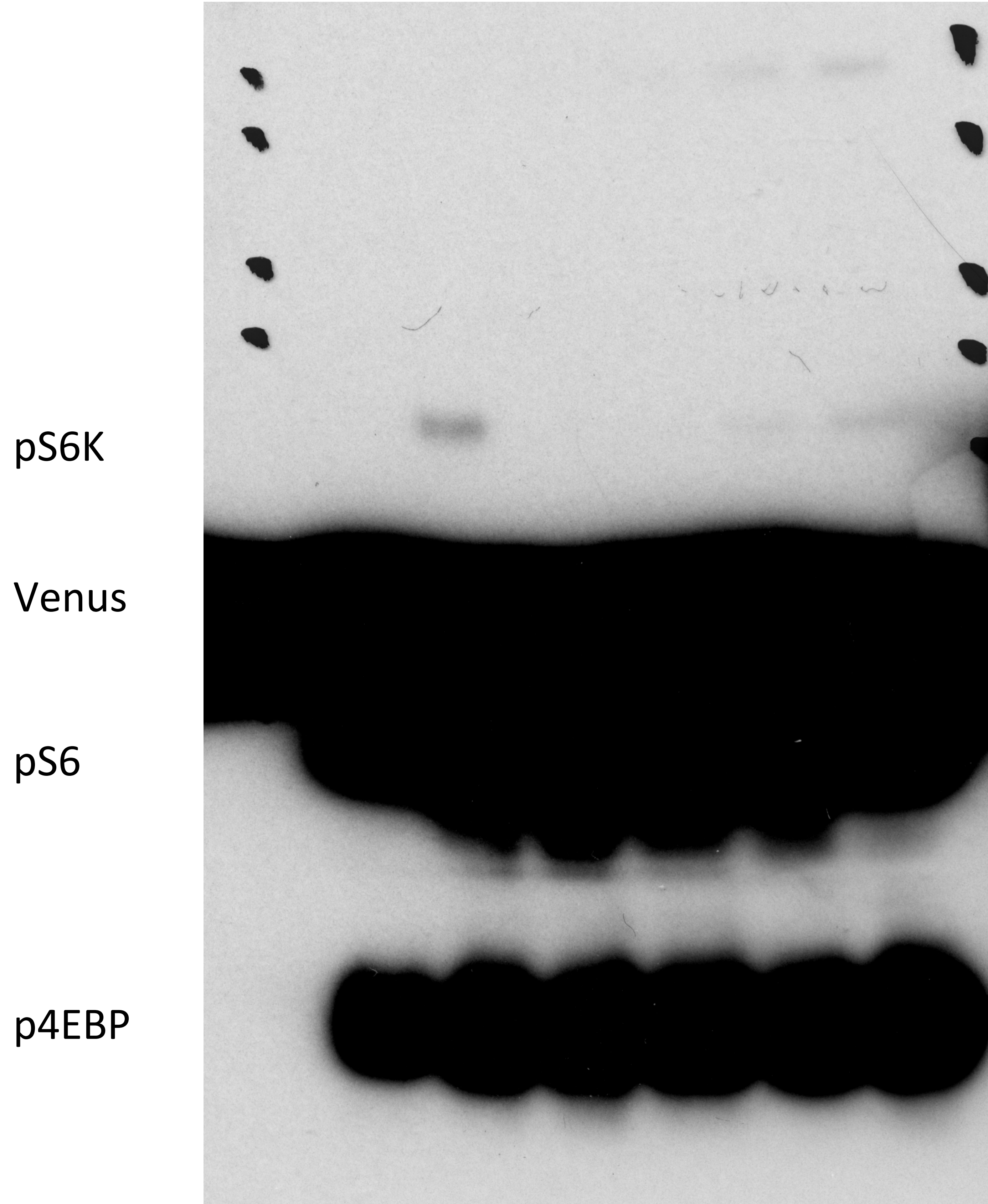

Gel 1: re-probe short exposure

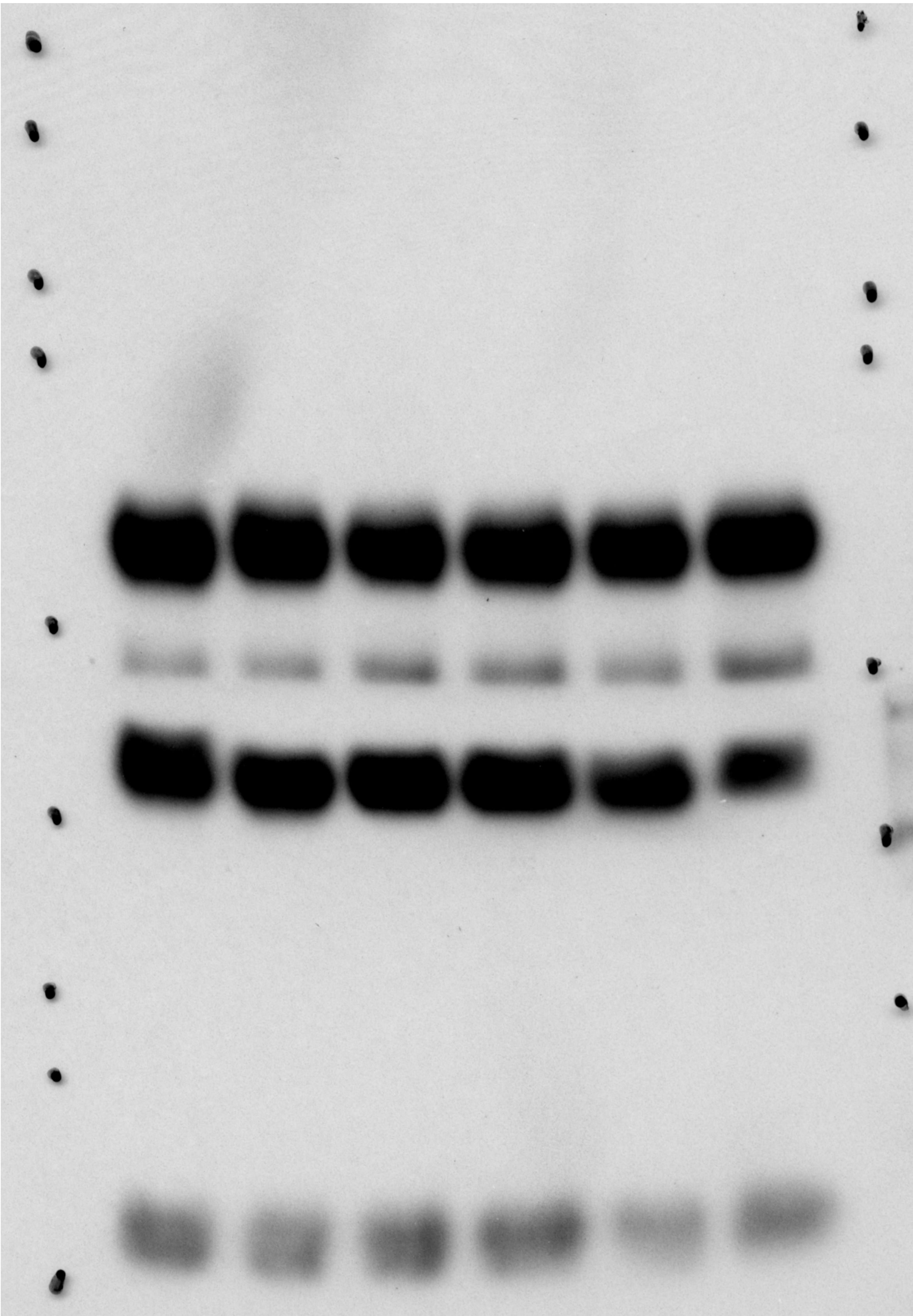

Gel 1: re-probe mid exposure

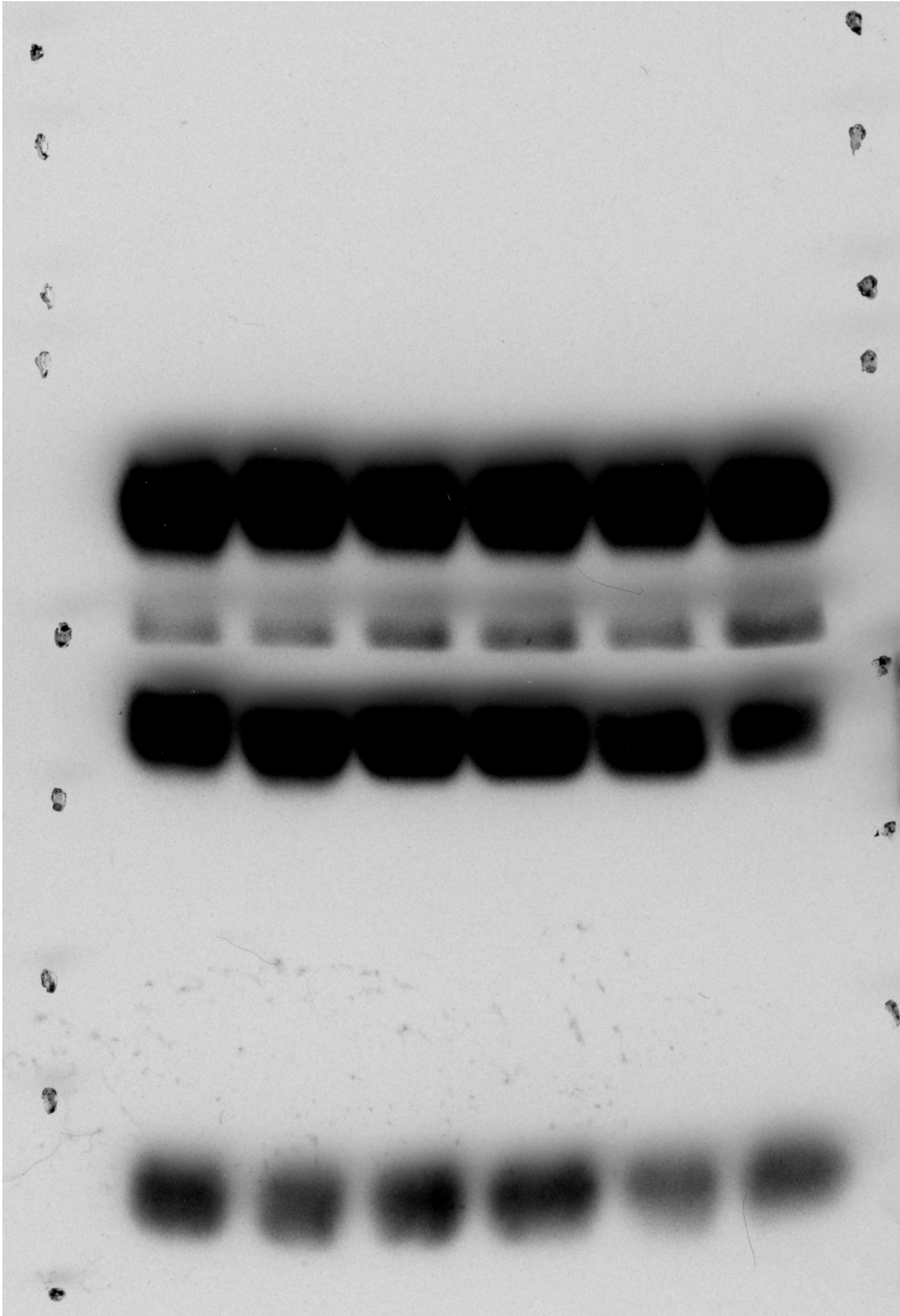

Gel 1: re-probe long exposure

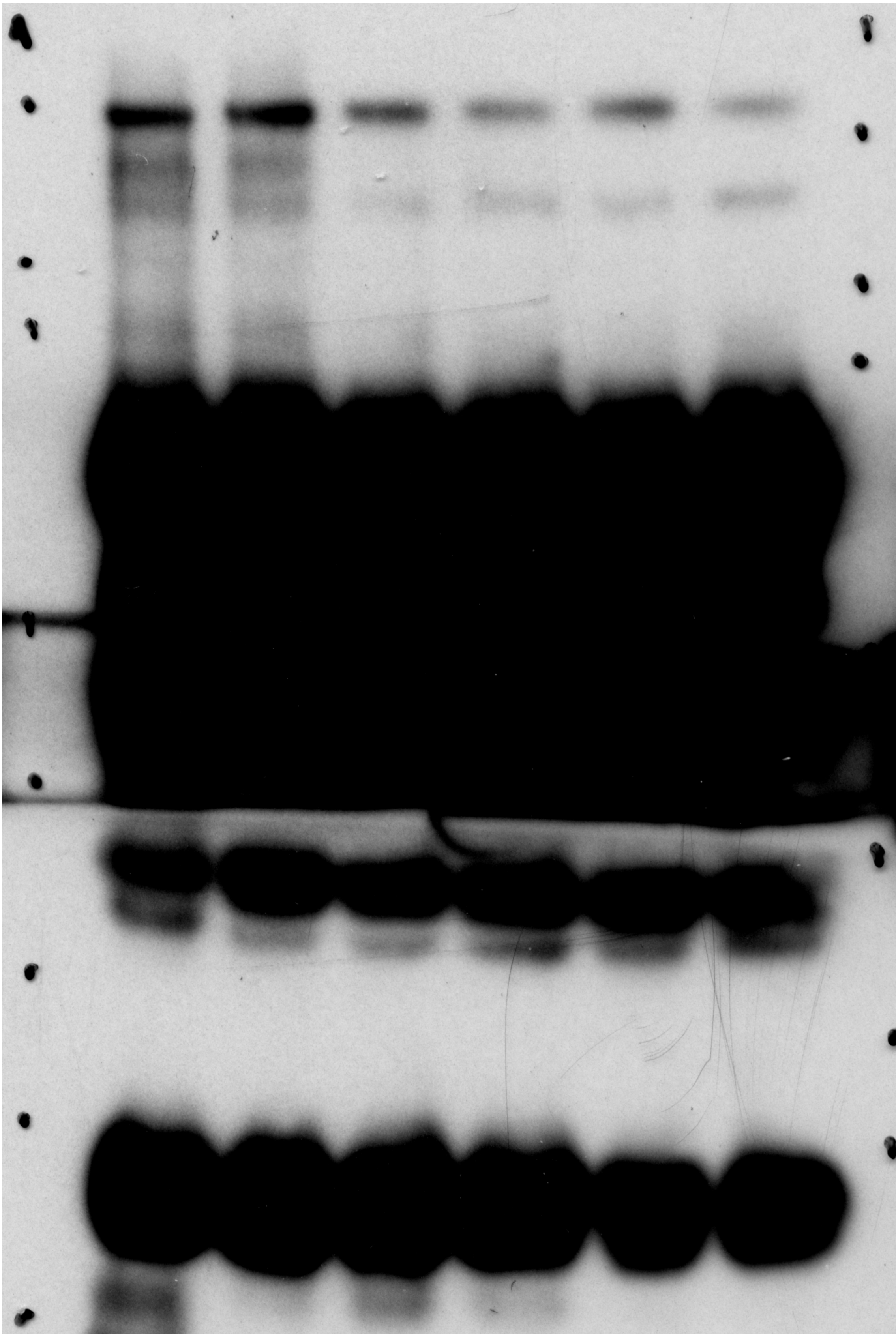

Gel 2:short exposure

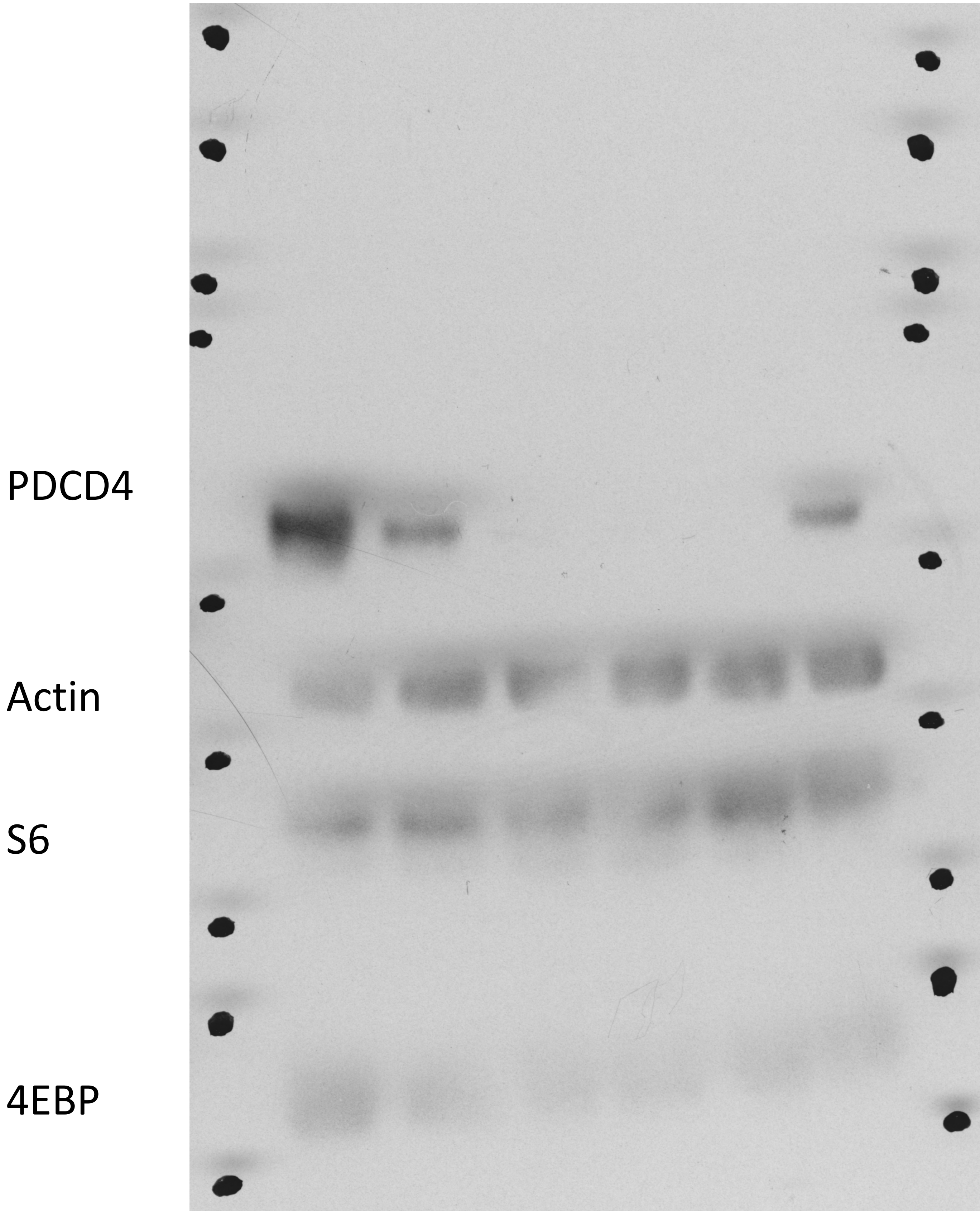

Gel 2:long exposure

PDCD4

Actin

S6

4EBP

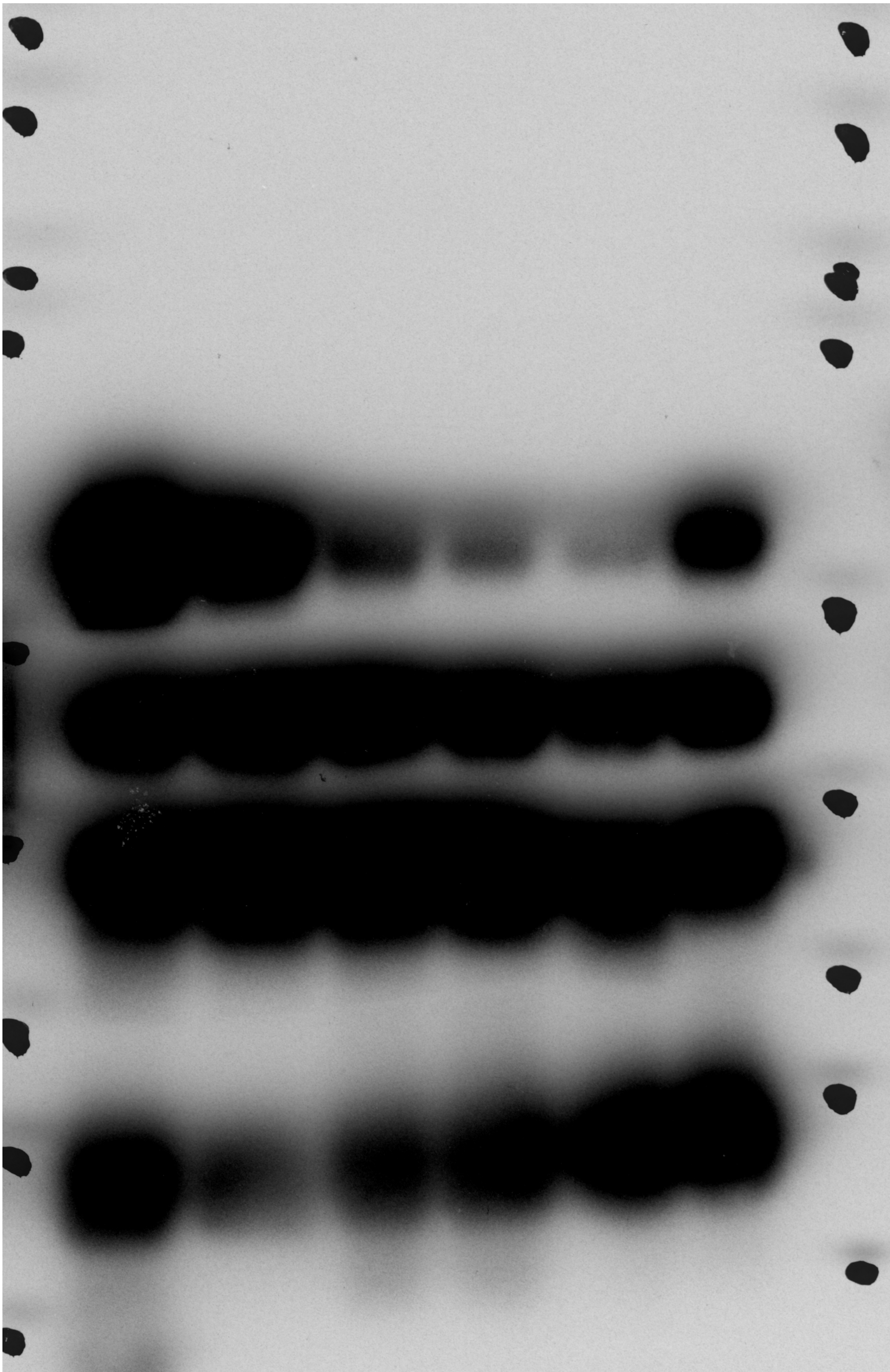

Supplement: Supplementary file 7 — Source Data [file 41467_2020_20491_MOESM7_ESM.zip › Source data/Western raw data.pdf]
